# How Stand Productivity Results from Size- and Competition-Dependent Growth and Mortality

**DOI:** 10.1371/journal.pone.0028660

**Published:** 2011-12-13

**Authors:** John P. Caspersen, Mark C. Vanderwel, William G. Cole, Drew W. Purves

**Affiliations:** 1 Faculty of Forestry, University of Toronto, Toronto, Canada; 2 Computational Ecology and Environmental Science Group, Microsoft Research, Cambridge, United Kingdom; 3 Ontario Forest Research Institute, Sault Ste. Marie, Canada; University of California, Berkeley, United States of America

## Abstract

**Background:**

A better understanding of the relationship between stand structure and productivity is required for the development of: a) scalable models that can accurately predict growth and yield dynamics for the world's forests; and b) stand management regimes that maximize wood and/or timber yield, while maintaining structural and species diversity.

**Methods:**

We develop a cohort-based canopy competition model (“CAIN”), parameterized with inventory data from Ontario, Canada, to examine the relationship between stand structure and productivity. Tree growth, mortality and recruitment are quantified as functions of diameter and asymmetric competition, using a competition index (*CAI_h_*) defined as the total projected area of tree crowns at a given tree's mid-crown height. Stand growth, mortality, and yield are simulated for inventoried stands, and also for hypothetical stands differing in total volume and tree size distribution.

**Results:**

For a given diameter, tree growth decreases as *CAI_h_* increases, whereas the probability of mortality increases. For a given *CAI_h_*, diameter growth exhibits a humped pattern with respect to diameter, whereas mortality exhibits a U-shaped pattern reflecting senescence of large trees. For a fixed size distribution, stand growth increases asymptotically with total density, whereas mortality increases monotonically. Thus, net productivity peaks at an intermediate volume of 100–150 m^3^/ha, and approaches zero at 250 m^3^/ha. However, for a fixed stand volume, mortality due to senescence decreases if the proportion of large trees decreases as overall density increases. This size-related reduction in mortality offsets the density-related increase in mortality, resulting in a 40% increase in yield.

**Conclusions:**

Size-related variation in growth and mortality exerts a profound influence on the relationship between stand structure and productivity. Dense stands dominated by small trees yield more wood than stands dominated by fewer large trees, because the relative growth rate of small trees is higher, and because they are less likely to die.

## Introduction

Plant ecologists have long sought to understand how the size-dependence of competition, growth, and mortality influences the structure and productivity of plant populations [1]–[7]. But there is a growing need to develop a much better understanding of these relationships for one special class of plant populations – forests. Forests harbor two thirds of terrestrial biodiversity [8] and half of terrestrial carbon [9], while also providing lumber, fibre, and fuel to humanity. These ecosystem services are threatened by climate and land-use change, so there is increasing urgency to develop models that can predict the ecological dynamics of the world's forests [10], [11]. Yet, it remains uncertain how size- and competition-dependent growth and mortality of individual trees scale to the population level to determine stand productivity.

The manner in which allometry, competition, and senescence together determine the relationship between stand structure and productivity has been a topic of research in ecology and forestry for some time [1]. However, this research has not lead to many generalities that can be applied broadly to different kinds of forests. Without a fundamental and generalizable understanding of this tree-to-stand scaling, it will be impossible to develop forest models that are sufficiently realistic to simulate a wide variety of ecological processes, whilst being sufficiently simple to implement at both local and global scales [10], [11].

At local scales, complex models have been used to simulate most of the ecological processes that drive stand dynamics. For example, spatially-explicit, individual-based models can predict the dynamics of succession because they simulate height-structured competition for light, as well as stochastic spatial processes such as tree mortality [12]. However, complex models are computationally intensive and therefore cannot easily be used to simulate forest dynamics at regional or global scales. Furthermore, these models require a large amount of detailed information (e.g. hemispherical photographs for estimating species-specific responses to light availability) that is not readily obtained at regional or global scales [13]. Perhaps more importantly, it is difficult to identify generalities in the behavior of complex models that (if only they could be identified) might be used to develop a more general understanding of forest dynamics, and to advance generalizable principles for forest management.

Conversely, simple models are designed to capture a few key processes thought to be the primary determinants of forest structure and function. For example, the metabolic theory of ecology (MTE) seeks to explain the power-law scaling relationships commonly observed at the level of individuals trees (such as that between tree size and growth rate), and uses simple models to examine the stand-level consequences of the underlying tree-level processes [14], [15]. This theory represents an important contribution to ecological knowledge, and the models are highly scalable because they are so simple. However, this same simplicity means that they are unlikely to provide an understanding of forest dynamics that is accurate enough to be useful for addressing important applied questions. For example, MTE models do not simulate resource competition (or any other form of interaction among individual trees), even though asymmetric competition for light is widely recognized as a key driver of forest dynamics [16]–[18], [15]. Thus, they are unlikely to provide insights into the relationship between stand structure and productivity that will improve growth and yield forecasts.

Toward this end, more empirically-oriented research has sought to determine whether uneven-aged stands are more productive than even-aged stands, either biologically [1], [19], [20] or economically [21]. Several studies have also compared the productivity of uneven-aged stands that differ in stand structure, including both the density and size distribution of trees [22]–[26]. However, these studies have not examined demographic processes in detail and, therefore, do not provide direct evidence for the mechanisms underlying variation in stand productivity.

It should be possible to steer a path between these different approaches by identifying the key processes that drive the dynamics of different kinds of forests, representing these processes parsimoniously within models, and constraining the models against empirical data to ensure that the models retain an appropriate fidelity to the dynamics of particular forests. One of us (DWP) was involved in an attempt to do this for forests structured primarily by competition for light, via the Perfect Plasticity Approximation (PPA) model [27], which focuses on height-structured competition for canopy space. The simplest version of this model reproduces successional dynamics of forests in the US Lake States [28] whilst also providing general insights about which species dominate early- and late-successional niches in disturbed landscapes [29]. However, this simple version ignores several key drivers, including interspecific variation in shading and in crown geometry. Most notably for understanding productivity, it does not allow for any size-related variation in the growth or mortality of canopy trees.

Evidently this variation is not crucial for understanding the 80 to 100-year trajectories of undisturbed stands studied by [28]. However, there is considerable evidence that it occurs, for example through senesecence and perhaps increased exposure to disturbance, whereby mortality increases as trees grow older and larger [30]–[32], [16]–[18]. Growth begins to decline at some point as well, such that that large trees are less efficient than smaller trees, producing less wood per square meter of crown area [1], [33]–[37]. Thus, size-dependence is likely to be key for understanding forest dynamics in the longer term (by which time there will be more large trees), and for understanding how management affects growth and yield through its effect on stand structure.

In this paper, we address the need to develop scalable models that can accurately predict, and provide a more general understanding of, the growth and yield dynamics of uneven-aged stands, including the relationship between stand structure and productivity. In order to do so, scalable models must realistically simulate the size asymmetry of competition for light [38], as well as size-dependent variation in growth and mortality, particularly that associated with senescence [18], [32]. Thus, we introduce a spatially-implicit cohort model (“CAIN”) that simulates height-structured competition for canopy space using size-dependent demographic parameters estimated from inventory data. We demonstrate that the model accurately predicts the growth and yield dynamics of partially harvested forests, while retaining the advantages of scalable models, including computational speed and ease of parameterization. Furthermore, we show that the model provides new insights into the relationships between stand structure and productivity, and reveals potential trade-offs between maximizing productivity and maintaining the structural and species diversity of managed forests.

## Methods

The CAIN model developed here is derived from the PPA model, which is also known as the Ideal Tree Distribution (ITD) model [39]. The initial paper describing the PPA model demonstrated that it accurately predicts crown and canopy structure, based on the density, diameter distribution and species composition of a stand [39]. Two subsequent papers showed that the canopy metrics produced by the PPA can be incorporated into dynamical models that accurately simulate both succession and coexistence [27], [28], even in the simplest version of the model where competition between canopy trees is assumed to be strictly size symmetric (all canopy trees have the same growth and mortality rate, regardless of size). In this paper, we relax size symmetry in order to simulate the differential suppression of canopy trees and its effect on the growth and yield of managed stands.

Below, we first describe the CAIN model, including the canopy-based metric (*CAI_h_*) that we use to quantify the size asymmetry of competition, as well as the equations used to predict demographic rates (allometric functions are described in Appendix S1). Then, we describe the inventory data and statistical techniques used to estimate the model parameters. Finally, we describe a series of simulations used to examine the relationship between stand structure and productivity.

The *CAI_h_* metric and functional forms described below were chosen to obtain predictions that matched all the available tree- and stand-level data, including the geometry of tree crowns, the diameter- and competition-dependence of tree growth and mortality, and the observed relationships between stand structure and productivity.

### Competition

We relaxed size symmetry by allowing the growth and mortality of each tree to depend on the height of its crown, as well as the projected area of all other crowns at that height. In particular, we allowed growth and mortality to vary as a function of the crown area index (*CAI*) at height *h*, the midpoint of the tree's crown (see Appendix S1). The crown area index at height *h* is
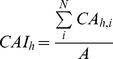
(1)where *CA_h,i_* is the projected area (m^2^) of crown *i* at height *h* (see Appendix S1), *N* is the number of trees in the stand, *A* is the area of the stand, and *CAI_h_* (m^2^/m^2^) is normalized by the area of the stand (Table 1). The crown area index at height *h* includes the projected crown area (at height *h*) of all trees taller than *h*. The projected area remains constant between the base of a crown and the ground (see Appendix S1), so *CAI_h_* is a cumulative metric that increases monotonically from the top to the bottom of the canopy.

**Table 1:** Description of parameters and variables.

Model	Equation	Parameter/variable	Description
**Height allometry (H)**	Appendix S1, eq. S1	η	Slope of the relationship between stem diameter and tree height
		ϕ	Asymptote of the relationship between stem diameter and tree height
**Crown depth (V)**	Appendix S1, eq. S2	ϖ	Ratio of crown depth to tree height
**Crown radius (R_h_)**	Appendix S1, eq. S4	β	Crown shape (1 = cone, 0 = cylinder)
		r_0_	Maximum crown radius at a stem diameter of 0 cm
		r_40_	Maximum crown radius at a stem diameter of 40 cm
**Crown area**	Appendix S1, eq. S7	CA_i,h_	Projected area of crown *i* at height *h* (the midpoint of the crown)
**Crown area index**	Equation 1	CAI_h_	Crown area index at height *h*: includes crown area of trees taller than *h*
**Growth (G)**	Equation 2	δ	Baseline growth rate
	Figure 1, panel A	γ	Location parameter of the log-normal multiplier (size effect)
		ν	Scale parameter of the log-normal multiplier (size effect)
	Figure 1, panel B	ζ	Minimum value of the negative exponential multiplier (competition effect)
		κ	Decay rate of the negative exponential multiplier (competition effect)
**Mortality (M)**	Equation 3	ψ	Baseline longevity
	Figure 1, panel C	φ	Exponent of the increasing power function (size effect)
		θ	Inflection point of the logistic function (size effect), expressed as a proportion of D_0.01_
		D_0.01_	The diameter at which logistic function takes a value of 0.01 (size effect)
	Figure 1, panel D	ω	Minimum value of the negative exponential multiplier (competition effect)
		o	Decay rate of the negative exponential multiplier (competition effect)
**Ingrowth (I)**	Equation 5	τ	Baseline ingrowth rate, if species is present
	Figure 1, panel E	χ	Minimum value of the negative exponential multiplier (competition effect)
		υ	Decay rate of the negative exponential multiplier (competition effect)
		CAI_0.05_	Lower 5^th^ percentile of *CAI_0_* values at which ingrowth is capped (competition effect)
	Equation 5	ξ	Baseline ingrowth rate, if species is absent
**Error distributions**	Appendix S3, eq. S13	σ_H_	Residual standard deviation of height
		σ_V_	Residual standard deviation of crown depth
		σ_W_	Residual standard deviation of crowth width
		ρ_HV_	Correlation between tree height and crown depth
		ρ_HW_	Correlation between tree height and crown width
		ρ_VW_	Correlation between crown depth and crown width
		σ_G_	Residual standard deviation of growth
	Appendix S3, eq. S16	Ω_P_	Overdispersion parameter when species is present
		Ω_A_	Overdispersion parameter when species is absent
**Stand effects (E)**	Equation 4	α	Scales stand effects for longevity
		π	Scales stand effects for ingrowth
**Stand error structure**	Appendix S3, eq. S17	σ_E_	Standard deviation of stand effects

### Demography

We modeled tree growth and mortality as the product of a baseline rate, a random stand-effect term (E), and two terms that account for the effects of tree size (S) and competition (C):
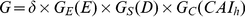
(2)

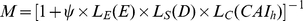
(3)where *G* is the annual diameter (*D*) growth rate, *M* is the annual mortality rate, and *δ* and *ψ* are species-specific parameters that the specify the baseline growth and longevity (*L*). Longevity (expressed in years) is the reciprocal of the annual mortality rate. For example, if the annual mortality rate (*M*) is 0.01, then longevity (*L*) is 100 years.

**Figure 1:**
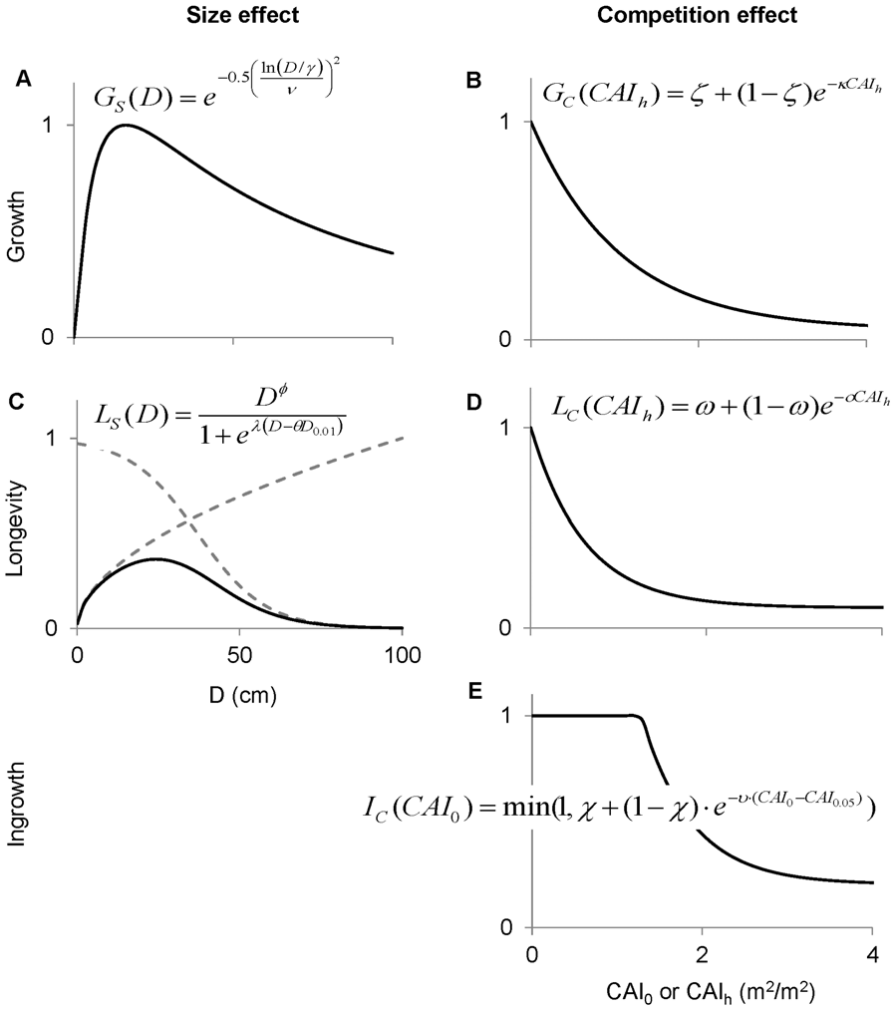
Equations and graphs illustrating the effect of tree size and competition on demographic rates. The equations relate diameter growth (top panels: A,B), longevity (middle panels: C,D), and ingrowth (bottom panel: E) to tree diameter (D; left panels: A,C) and crown area index (*CAI_h_* and *CAI_0_*; right panels: B, D). The effect of diameter on longevity (*L_S_(D)*, middle left) is specified as the product of an increasing power function and a decreasing logistic function (grey dashed lines). To aid with model fitting, we reparameterized the slope (λ) of the logistic function so that it takes a value of 0.01 at diameter *D_0.01_*, and the inflection point is expressed as a proportion (*θ*) of *D_0.01_*, using the relationship *λ = ln(99)/(D_0.01_·(1−θ))*. Ingrowth is capped at the maximum rate when crown area index is lower than *CAI_0.05_*, the lowest 5^th^ percentile of observed *CAI_0_* values in plots where the species is present. Including this 5^th^-percentile cap avoids overestimating recruitment in open stands, for which there were few observations. All functional forms were plotted using parameter values for sugar maple (Table 1).

To account for random stand effects, the stand-specific parameter *E* quantifies the unexplained tendency for trees within a stand to show systematically higher or lower growth and longevity than the mean across all stands:
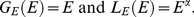
(4)To account for the effect of tree size, growth (*G_S_(D)*) varies as a log-normal function of diameter [40], whereas longevity (*L_S_(D)*) both increases as a power function of diameter and decreases as a sigmoidal function of diameter to produce an overall U-shaped mortality function [31], [32]. To account for the effect of competition, both growth and longevity (*G_C_(CAI_h_)*, *L_C_(CAI_h_)*) decrease as negative exponential functions of *CAI_h_*. Equations and graphical illustrations of the functional forms are presented in Figure 1.

We modeled annual recruitment (#/ha/yr), or ingrowth, of new stems (those reaching 2.5 cm in diameter) of each species as the product of a baseline rate (which depends on whether the species is already present), a random stand-effect term, and the crown area index of the stand (if the species is present):
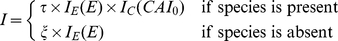
(5)where *I* is the ingrowth rate, *τ* and *ξ* are baseline rates, and *I_E_(E)* = *E^π^*. Like growth and mortality, ingrowth (*I_C_(CAI_0_)*) decreases as a negative exponential function of crown area index (Figure 1).

### Study area

The model was parameterized for the Great Lakes – St. Lawrence forest region [41] of central Ontario, Canada (44–48°N, 74–85°W). Tolerant hardwood forests in this region are generally dominated by sugar maple (*Acer saccharum* Marsh.), in association with beech (*Fagus grandifolia* Ehrh.), yellow birch (*Betula alleghaniensis* L.), eastern hemlock (*Tsuga Canadensis* (L.) Carr.), red maple (*Acer rubrum* L.), white ash (*Fraxinus Americana* L.), basswood (*Tilia Americana* L.) and ironwood (*Ostrya virginiana* (Mill.) K.Koch).

Tolerant hardwood forests in central Ontario are typically managed using single-tree selection [42], in which a portion of the stand is harvested every 15–25 years. Thus, the structure of selection-managed stands is determined by harvest targets specifying the total volume (or basal area) of residual trees, as well as their distribution across different size classes [43]. These harvest targets are chosen in the attempt to maximize the yield of timber (merchantable logs exceeding a minimum diameter), so it is not known whether they also serve to maximize the total yield of wood, including small trees that cannot be sold as lumber, but may have value in emerging bioenergy and carbon markets.

### Inventory data

The model was parameterized with three inventory datasets collected by the Ontario Ministry of Natural Resources. Of the three datasets, the Permanent Sampling Plot (PSP) dataset is the most extensive, including 280 plots located throughout central Ontario, Canada (44–48°N, 74–85°W) across an area of approximately 110,000 km^2^. The 0.04 ha plots are circular (radius = 11.28 m), and were established in groups of three within stands. All trees ≥2.5 cm in diameter at breast height were measured twice between 1993 and 2001, with a median interval of 5 years. The standard inventory data recorded for each plot include diameter at breast height (*D*), species identity, and status (*M_o_*: live = 1, dead = 0). Further details about the PSP dataset are provided in [44] and [45].

The Algonquin Region (AR) dataset is less extensive, including 251 plots located in the Algonquin region of central Ontario (45–46°N, 77–79°W), spanning an area of approximately 10,000 km^2^. The 0.04 ha plots are square (20 m×20 m), and all trees ≥9.1 cm in dbh were measured in 1977 and 1982. In addition to the standard inventory data listed above, several other observations were also recorded, including tree height (*H_o_*), crown depth (*V_o_*), and crown diameter or width (*W_o_*), as measured on a single representative axis. Further details about the AR dataset are provided in [46].

The Parkside Bay (PB) dataset was collected at a single site (45°N, 79°W) within Algonquin Park, and includes 56 plots spanning an area of approximately 1 km^2^. The 0.04 ha plots are square (20 m×20 m), and all trees ≥5.0 cm in dbh were inventoried in 1995 and 2000. In addition to the standard inventory data listed above, tree height and crown depth were measured using the same methods used for the AR dataset. Crown radius was also measured, but it was measured in each of four cardinal directions. Further details about the methods are provided in [47].

### Parameter estimation

Since crown measurements were not taken in the PSP dataset, we estimated model parameters in three steps as follows. First, we estimated the crown parameters by fitting the crown models (Appendix S1) to the crown data (*H_o_*, *V_o_* and *W_o_*) from the PB and AR datasets. Second, we used the fitted crown models to calculate crown and canopy metrics (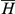, 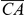, 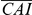 and 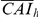) for each tree and plot in all three datasets, including the PSP dataset. Third, we estimated all of the remaining parameters by fitting the demographic models to all three datasets, using 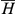, 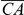, 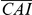 and 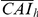 as predictor variables in equations 1–9. To account for random stand effects, unique values of *E* were estimated for each individual plot in the AR dataset, for each group of three plots in the PSP dataset, and for the complete set of 56 plots in the PB dataset. The maximum likelihood methods used to estimate all parameters are described in Appendix S2 and S3.

### Model simulations

To examine the relationship between stand structure and productivity, we simulated growth, mortality, and yield for each inventoried stand, as well as 15 hypothetical stands that differed in total volume and the size distribution of trees. For the hypothetical stands, the simulations were initialized by specifying the wood volume of the stand, and the density of trees in each 5 cm diameter class between 5 and 60 cm diameter. The maximum diameter was set to 60 cm because the provincial guidelines for the Algonquin region recommend that all trees greater than 60 cm be removed during selection harvests [43]. Also in keeping with silvicultural conventions, the diameter distribution of residual stands was specified using a *q* ratio, the ratio of the number of trees between successive 5-cm diameter classes [43]. When *q* is one, the diameter distribution is uniform: when *q* is greater than one, diameter follows a negative exponential distribution. As *q* increases, the total density of trees increases and the average size decreases. Also note that for a given value of *q*, the density in each size class scales with the total volume: for example, doubling the total stand volume, is achieved by doubling the density in each size class.

We chose harvest targets to span a broad range of stand structures representative of uneven-aged stands: across the 15 simulations, the target volume ranged from 50–250 m^3^/ha, and the *q* ratio varied from 1.3 to 1.9. We held stand composition relatively constant by specifying the abundance of species within each 5-cm diameter class, using the relative abundances observed in all three datasets (Table S1). Once the diameter distribution and species composition were initialized, we simulated stand dynamics over twenty years (a typical rotation interval under selection silviculture) and calculated total growth, mortality, and yield for each of the simulated stands.

There was modest variation in stand composition across the simulations due to the uneven distribution of some species across size classes. For example, iron wood was absent from the largest size classes (Table S1), so its relative abundance decreased as *q* decreased. To assess whether this modest variation had any effect on the simulated growth and yield, we compared the 15 simulations described above to 15 equivalent simulations of sugar maple monocultures. However, the monoculture simulations differed little from the mixed species simulations, so we do not report on them below.

## Results

We were able to estimate all of the model parameters for sugar maple, and most of the model parameters for seven other species (Table S2). For these seven species there were too few large trees to reliably estimate the mortality parameter 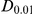, the diameter at which logistic function takes a value of 0.01 (Fig. 1). Thus, we fixed 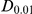 at the maximum observed diameter of each of the seven species to ensure that they had a minimal probability of survival upon reaching their largest observed size. For all other parameters, we report the maximum likelihood estimate (Table S2) with confidence limits (Table S3; for goodness-of-fit, see Figures S1, S2, S3, S4, S5, and S6).

### Demography

**Figure 2:**
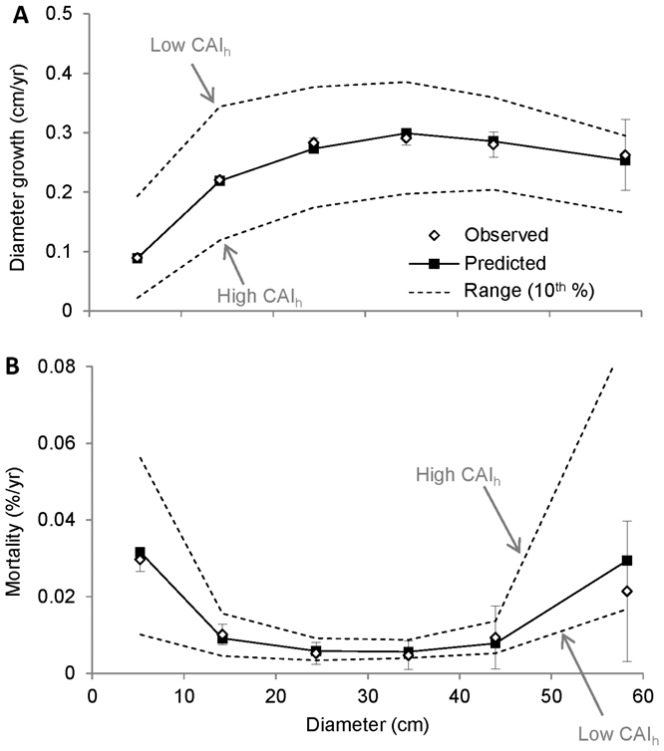
Size dependence of growth and mortality. The growth (A) and mortality (B) data were sorted into six diameter bins (<10, 10–20, 20–30, 30–40, 40–50, >50) and the mean growth and mortality was calculated for each bin. Error bars show ±2 s.e. for observed growth and mortality. For each bin, the predicted range (dashed lines) was calculated by ranking each tree by *CAI_h_*, then calculating the average predicted growth and mortality for the top and bottom ten percent of values.

The parameter 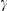 is the diameter at which a species is estimated to reach its maximum rate of diameter growth, all else (i.e. the amount of shading) being equal. For all but two species, 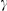 was less than 20 (Table S2), indicating that poles (trees 10–25 cm in diameter) tend to grow faster in diameter than larger trees when under equivalent shading. However, poles are likely to be shaded by larger trees, so the average growth rate of poles in the dataset (0.23 cm/yr) was less than the average growth rate of larger canopy trees (0.29 cm/yr) (Figure 2A).

The average growth rate of saplings (trees <10 cm in diameter) was even lower (0.08 cm/yr), because they generally occur in the understory (Figure 2A). However, the predicted growth rate of saplings varied by an order of magnitude (Figure 2A), from 0.2 cm/year to 0.02 cm/year (dashed lines). This reflects the wide range of variation in *CAI_h_* for saplings, which may be found in recently disturbed stands as well as the understory of mature stands. In contrast, the predicted growth rate of canopy trees only varied by a factor of two (Figure 2A), because they cannot be completely overtopped by competitors.

Mortality exhibited a broad U-shaped pattern with respect to diameter (Figure 2B). For saplings, the average mortality rate was 3% per year (Figure 2B), but the predicted probability of mortality varied from 1% per year to 6% per year (dashed lines), again reflecting the wide range of variation in *CAI_h_* for saplings. For trees 25–35 cm in diameter, the average probability of mortality was only 0.5% per year, because they cannot be overtopped by competitors (and thus have a lower average *CAI_h_*). Large canopy trees (>50 cm in diameter) also cannot be overtopped, but show mortality greater than 2% per year. This U-shaped mortality pattern has major implications for understanding the relationship between stand structure and productivity, because large trees contain a disproportionate fraction of stand volume, such that any increase in their mortality has a large effect on yield.

**Figure 3:**
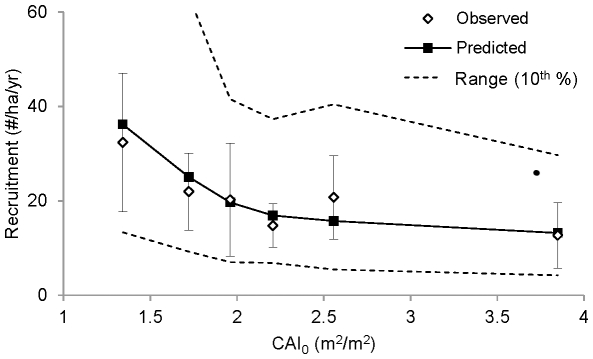
Recruitment in relation to crown area index (*CAI*_0_). The error bars show ±2 s.e. for the observed recruitment rate. The data were binned and averaged as in Figure 1, but in this case the bin widths were chosen to obtain an equal number of observations in each bin. For each bin, the predicted range (dashed lines) was calculated by ranking each plot by the predicted recruitment rate, then calculating the average recruitment rate for the top and bottom ten percent of values.

The crown area index (*CAI_0_*) of stands in the PSP dataset varied from 0.5 m^2^/m^2^ to over 6 m^2^/m^2^. The lower 5^th^ percentiles (*CAI_0.05_*), which bound modeled ingrowth rates, ranged from 1.3–1.5 m^2^/m^2^ for stands in which each species was present (Table S2). Over the range of *CAI_0_* values, the total predicted recruitment rate decreased about threefold, from 32 to 11 total recruits/ha/year (Figure 3). The average size of new recruits was 3.0 cm in diameter, 0.5 cm larger than the minimum sized tree inventoried in the PSP dataset.

Comparing species growth rates under low and high crown shading, relative decreases in growth ranged from 7–59% among the eight species, and closely followed an established shade tolerance gradient (Table 2). The four least shade tolerant species also had greater mortality rates under high shading than the four most shade tolerant species (3.0–10.9% vs. 0.3–2.6%), with the two most shade tolerant species (beech and eastern hemlock) showing particularly high survival in well-shaded conditions. Similarly, beech showed the smallest relative decrease in recruitment rate under high shading (20%), whereas three out of the four least shade tolerant species were reduced to less than one-third of their low-shading recruitment. Thus, changes in growth, mortality, and recruitment predicted for different *CAI_h_* values all correlated well with an established gradient in shade tolerance.

**Table 2:** Difference in mean predicted growth, mortality, and recruitment rates under low and high crown shading (*CAI_h_*<2, ≥2) for 8 species ranked by shade tolerance, following Baker (1949): intermediate (3), tolerant (4), very tolerant (5).

Rank	Species	Mean predicted demographic rates					
		Growth* (cm/yr)		Mortality* (/yr)		Recruitment† (#/ha/yr)	
		*CAI_h_*<2	*CAI_h_*≥2	*CAI_h_*<2	*CAI_h_*≥2	*CAI_h_*<2	*CAI_h_*≥2
3	White ash	0.167	0.069	0.016	0.030	3.401	1.775
3	Yellow birch	0.211	0.087	0.014	0.056	8.695	1.787
4	Red maple	0.191	0.102	0.023	0.109	2.566	0.532
4	Basswood	0.114	0.068	0.038	0.058	5.951	1.852
5	Sugar maple	0.141	0.075	0.017	0.026	15.902	7.462
5	Ironwood	0.082	0.059	0.008	0.011	12.330	6.319
5	Beech	0.108	0.074	0.007	0.008	3.559	2.822
5	Eastern hemlock	0.141	0.131	0.003	0.003	4.018	2.209

*Only including trees 5–9.9 cm DBH.

† Only including plots where species is present.

### Inventoried stands

**Figure 4:**
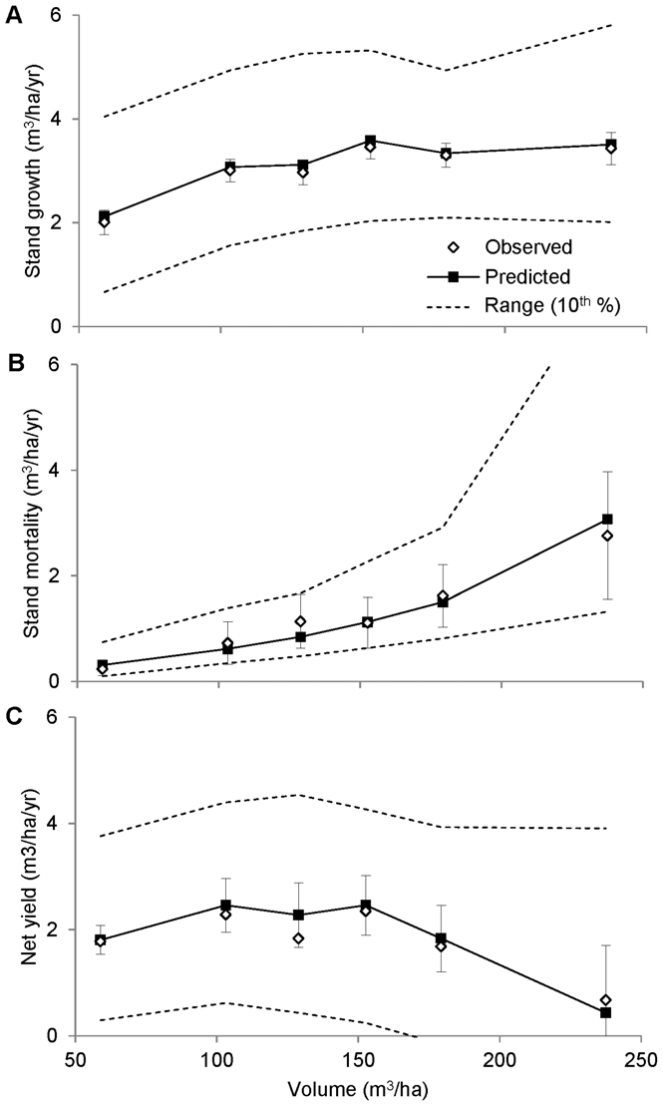
Growth, mortality, and yield of inventoried stands in relation to stand volume. The growth (A), mortality (B) and yield (C) data were binned and averaged as in Figure 1, but in this case the bin widths were chosen to obtain an equal number of observations in each bin. Error bars show ±2 s.e. for observed stand growth, stand mortality, and net yield. For each bin, the predicted range (dashed lines) was calculated by ranking each plot by the predicted rate, then calculating the average rate for top and bottom ten percent of values. Stands with trees >75 cm in diameter (*n* = 12) were excluded because of an overwhelming influence of very large trees on stand volume.

Stand volume varied from less than 50 m^3^/ha to more than 250 m^3^/ha. Across this range, observed stand growth increased from 2.0 to 3.5 m^3^/ha/year, leveling off at a volume of 150 m^3^/ha (Figure 4A). This asymptotic pattern was reproduced by the model because predicted growth initially increases with stand density, but then saturates as competition (reflected in increased *CAI_h_* for individual trees) offsets any further increases in density (Figure 4A).

The increase in growth was also offset by a compensatory increase in mortality (Figure 4B), a trend that was predicted by the model as well. In contrast to stand growth, the increase in mortality across the inventoried plots is monotonic, reflecting an increase in density (and therefore *CAI_h_* values) and average tree size, both of which contribute to the increase in mortality. The combination of asymptotic growth and monotonic mortality means that net volume increment exhibits a unimodal pattern that peaks between 100 and 150 m^3^/ha (Figure 4C), first increasing as stand growth increases faster than mortality, then decreasing as stand growth saturates and mortality continues to increase. Net volume increment approaches zero at 250 m^3^/ha, at which point stand growth is completely offset by mortality.

### Hypothetical stands

**Figure 5:**
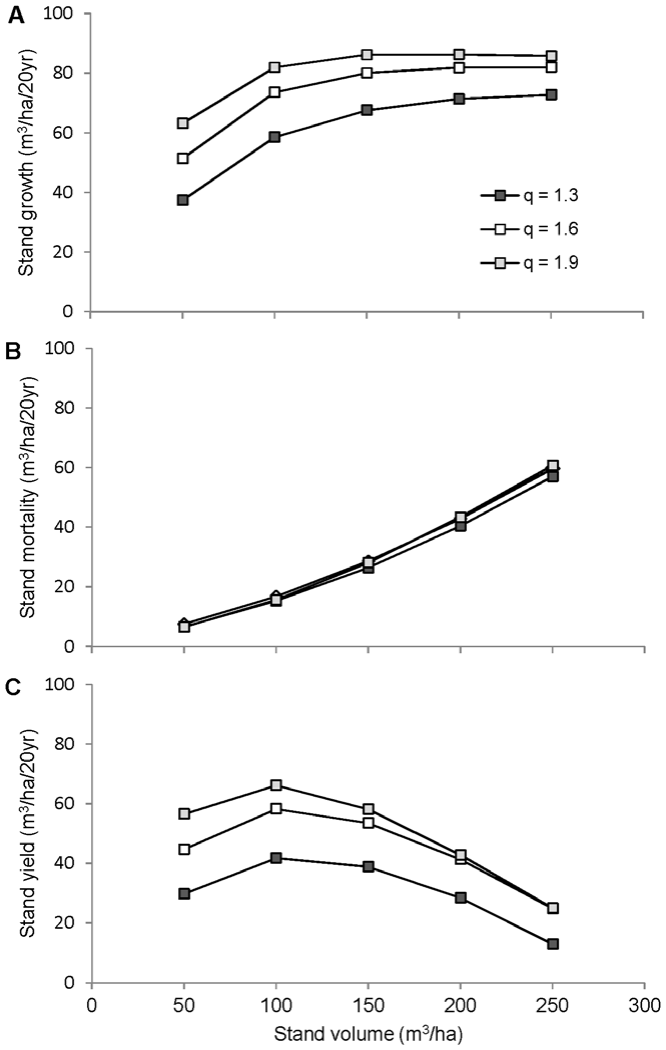
Simulated growth, mortality, and yield of hypothetical stands. The simulated stands differ in volume (x-axis) and size distribution (q), as well as growth (A), mortality (B), and yield (C).

The simulations in Figure 5 exhibit the same trends with respect to volume as observed for inventoried plots in Figure 4, but with an important difference. In Figure 4 (inventoried stands), the increase in volume from left to right reflects an increase in both stand density and average tree size, both of which covary with total stand volume. In Figure 5 (hypothetical stands), the increase in volume from left to right reflects an increase in density alone; for a given value of *q* the average tree size remains the same.

**Figure 6:**
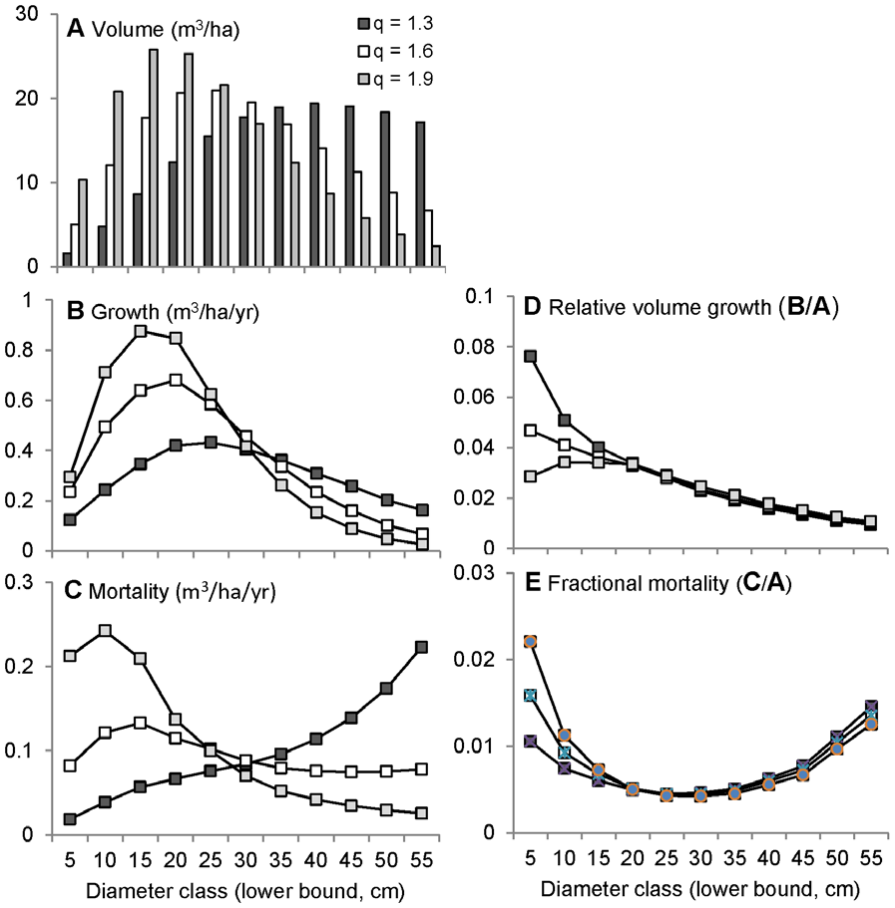
Simulated growth and mortality by size class. The distribution of volume (A), growth (B,D) and mortality (C,E) across size classes for three hypothetical stands with equal volume (150 m^3^/ha) but different size distributions (q).

As *q* increases, the total density of trees increases and the distribution of volume moves away from the larger size classes (Fig. 6A). The simulations show that increasing *q* results in a ∼40% increase in stand growth (Figure 5A). Dense stands are more productive because the additional trees provide added growth despite the heightened competition (higher *CAI_h_* for each tree). Decreasing the proportion of large trees also increases growth because the relative growth rate of large trees is less than that of smaller trees (Figure 6D).

Increasing density alone increases competition and hence stand mortality, as evident in the fact that mortality increased with volume (Figure 5B). However, increasing density by increasing *q* does not increase mortality (Figure 5B), because the proportion of large trees decreases (Figure 6A), and mortality rate of large trees is greater than that of smaller trees (Figure 6E). Thus, there is no net increase in mortality because the size-related reduction in mortality offsets the density-related increase in competition and attendant mortality.

The fact that increasing growth by increasing *q* (Figure 5A) does not result in a net increase in mortality (Figure 5B) explains why yield also increases by ∼40% (Figure 5C). In contrast, the increase in growth gained by increasing volume (Figure 5A) does incur a net increase in mortality (Figure 5B). Thus, the highest-yielding stand is obtained by decreasing the proportion of large trees while retaining at least 100 m^3^/ha of volume (Figure 5C). As explained above, maintaining an intermediate stand volume serves to maximize yield because growth increases asymptotically with volume (density), whereas mortality increases monotonically.

To provide further insight into the relationship between stand structure and productivity, we can examine the distribution of growth and mortality across size classes (Figure 6B, 6C). The contribution of different size classes to overall stand growth and mortality varies with *q* (Figure 6B, 6C), reflecting both stand structure (Figure 6A) and the differential allometric scaling of growth and mortality (Figure 6D, 6E). Since the relative volume growth rate declines with diameter (Figure 6D), volume growth in the large size classes (Figure 6B) is less than would be expected if growth were proportional to volume (Figure 6A). In contrast, fractional mortality (Figure 6E) exhibits a broad U-shaped pattern with respect to diameter, so volume loss in both the largest and smallest size classes (Figure 6C) is greater than would be expected if mortality were proportional to volume (Figure 6A). Due to this differential allometric scaling, the skewed distribution of mortality is reversed as *q* increases, but that of growth is not: smaller trees account for most of the stand growth, regardless of stand structure.

## Discussion

Our results provide three general insights into the growth and yield dynamics of uneven-aged stands. First, size-related variation in growth and mortality exerts a profound influence on the relationship between stand structure and productivity, even when the stands being compared exclude large, senescent trees (>60 cm in diameter). Second, to maximize wood yield uneven-aged stands should be managed to maximize the difference between growth and mortality for the stand as a whole by manipulating both total volume and its distribution across tree diameter classes. Third, dense stands dominated by small trees yield more wood than stands dominated by fewer large trees, both because the relative growth rate of small trees is higher, and because they are less likely to die.

Many previous studies have examined the size-dependence of growth and mortality [30], [31], [16], [17], [32], [33] and they broadly conform to our finding of hump-shaped growth and U-shaped mortality relationships with tree diameter. Several studies [34]–[37] have also shown that small trees are more efficient than large trees (consistent with Fig. 6D), and a few have examined how size-related variation in growth efficiency influences the relationship between stand structure and stand growth [20], [36]. However, none of these studies have examined how size-related variation in tree mortality influences the relationship between stand structure and mortality. Thus, they provide somewhat limited insight into the relationship between stand structure and yield.

For example, we have shown that increasing stand density while decreasing the proportion of large trees results in a 40% increase in stand growth (Figure 5A). Without any additional knowledge about the relationship between stand structure and mortality, this result might lead one to conclude that increasing productivity by 40% will also serve to increase yield by a similar amount. On the other hand, one might also expect that increasing stand density would result in a compensatory increase in mortality, thereby offsetting the increase in productivity. Thus, it is difficult to infer how stand structure influences yield without additional information on mortality.

Our mortality results show that there is, in fact, no such compensatory increase (Fig. 5B) because the proportion of large trees decreases (Figure 6A), and mortality rate of large trees is greater than that of smaller trees (Figure 6E). Thus, yield does increase if the proportion of large trees decreases as overall density increases (Fig. 5C), because the size-related reduction in mortality offsets the density-related increase in competition and attendant mortality. This result is consistent with those of Hansen and Nyland [24], who found that yield in sugar maple stands can be increased by about 25%, either by increasing *q* from 1.2 to 1.8, or by reducing maximum diameter from 60 cm to 40 cm. However, they did not evaluate the relative contribution of growth and mortality to increases in yield, and therefore do not provide a general understanding of why yield varies with the size distribution of trees.

We also find that stand dynamics are particularly sensitive to the shading of the smallest trees. Trees less than 10 cm in diameter tend to have relatively low diameter growth and high mortality, but both of these vital rates can vary greatly depending on the projected area of tree crowns above them (Figure 2). This pattern creates a strong negative feedback where well-shaded understory trees tend to remain small for a longer time, which considerably increases their chance of dying before growing into the canopy. As a result, there are fewer trees growing into the canopy to replace those that die (at increasing rates due to senescence), which in turn reduces the shade experienced by the understory trees.

Stand dynamics are also particularly sensitive to species differences in shade tolerance. While this was not the focus of our study, changes in growth, mortality and recruitment (Table 2) follow an established gradient in shade tolerance [48], [49], and are consistent with a trade-off between high-light growth and low-light survival among species which has been shown to drive succession [50]. In long-term, multi-species simulations with our model (not shown), changes in relative abundance through time correspond to species' ability to perform well either in young, open stands (shade intolerant, early successional species) or in older, more deeply shaded stands (shade tolerant, late successional species), in the absence of disturbance. Although such dynamics have been simulated before using complex, spatially explicit models [50], it is notable that our spatially implicit model can do so using an intuitive and easily calculated metric (*CAI_h_*) to quantify the asymmetry of competition for light, and that the data needed to recover these patterns can be obtained from forest inventories.

### Managing uneven-aged stands for wood yield and biodiversity conservation

When interpreting our results in practice, it is important to recognize that there are other economic and ecological objectives that may not be met by increasing productivity and maximizing the total yield of wood. Large trees are more valuable as timber (per unit wood volume) than small trees, because they are more likely to contain a merchantable sawlog [24]. Thus, decreasing the proportion of large trees may not increase timber yield, even though it increases total volume yield. On the other hand, emerging bioenergy markets may create demand for small logs, and thus economic incentives for increasing total volume yield. Increasing productivity and yield could also increase the amount of carbon sequestered in forests and wood products.

However, reducing the number of large trees may conflict with conservation objectives. While less productive and prone to death, large tree also generate structural heterogeneity, both aboveground and on the forest floor. This structural heterogeneity, in turn, helps maintain biodiversity by providing habitat or regeneration niches for particular species.

On the forest floor, downed woody debris provides critical habitat for ground-dwelling animals, insects and fungi [51]–[55], as well as a regeneration substrate for various tree and herb species [56]. Thus, retaining large trees that are likely to die helps maintain biodiversity by ensuring a continued supply of deadwood to the forest floor [51], [52], [54], [55], [57], [58].

Large trees also provide critical wildlife habitat above ground, particularly for birds and mammals that prefer to feed and nest in the cavities of large, declining trees and large, well-decayed snags [52], [59]–[62]. Thus, retaining large trees that are likely to die (and to be well-decayed when they do) helps to maintain biodiversity by ensuring a continued supply of suitable nesting cavities and feeding sites [52], [57], [58], [61], [62].

To provide better guidance to forest managers, the various benefits of retaining large trees should be quantified using a common set of metrics [58], then weighed against the resulting reductions in wood yield. Simulations similar to those presented here, but which focus on relationships between stand structure and species diversity (e.g. how the number and diversity of nesting birds depends on the number of large trees or snags), could provide a rigorous assessment of the trade-offs between maximizing wood yield and maintaining biodiversity [58].

### Modelling growth and yield using cohort-based canopy models

Many forest models capture the asymmetry of competition by simulating resource use explicitly. For example, three-dimensional ray-tracing algorithms are used to predict light interception as a function of sun angle and the height and spatial arrangement of individual trees [63].

The advantage of this approach is that it realistically simulates the geometry of light interception and thus the extent to which height differences between trees determine disparities in growth suppression. However, light competition models are computationally intensive, and are not widely used to predict growth and yield because they cannot be calibrated directly from forest inventory data [13].

An alternative approach to simulating asymmetric competition is to use neighborhood models to directly predict growth and/or mortality as a function of the proximity, species, and relative size of individual competitors [64]. While such models can be calibrated with forest inventory data, they are also computationally intensive, and separate parameters must be estimated for each pair of species. Thus, they cannot be readily used to simulate forest dynamics at regional or global scales.

In this paper, we have developed a scalable model for simulating asymmetric competition that is both conceptually intuitive and easy to parameterize. The model is intuitive because crown area index is a simple concept with a tangible physical interpretation, and because it is easy to understand how the growth and mortality of a tree depends both on its height within the canopy and the crown area index of competitors at that height. The model is easy to parameterize because doing so relies solely on inventory data, though many forest inventories may not include sufficient data to estimate all of the mortality or crown parameters.

While inventories are designed to quantify demographic rates, crown area and height are not standard forest inventory variables. However, enhanced forest inventories often include both, so the data required to estimate all the parameters are available for extensive areas [39]. Where enhanced inventories are not available, a targeted field campaign could be conducted to estimate the crown parameters using supplementary height and crown measurements taken on a subset of plots in a forest inventory. The fitted crown models could then be applied to all the plots in the inventory to estimate the demographic parameters. This approach would be conceptually similar to the parameterization method proposed by Lichstein *et al.* [13], who used supplementary light measurements taken above inventoried saplings to parameterize a model that can predict light availability for each sapling in a plot, using standard inventory measurements.

### Modeling forest dynamics at regional and global scales

Forest inventory data are available for most of the world's major forest types, including tropical forests in Africa, Asia, and the Americas [65], [66]. There is considerable potential for calibrating regional and global models using these inventory data in conjunction with supplementary datasets, including crown or resource measurements [13], [39], as well as plant trait databases for quantifying the relationships between functional traits and vital rates [67]. Yet, this potential has not been fully realized because it is challenging to estimate a full suite of life-history parameters for a multitude of species.

Recent efforts have confronted the need to a) strike a balance between model fit and model parsimony (maximizing the amount of variation explained while minimizing the number of parameters); b) capture the multidimensional life-history variation among species; c) include rare species for which there are few observations; and d) integrate trait-rate relationships to leverage supplementary datasets and thereby address challenges a-c above. For example, Purves et al. [39] developed a method for reducing multidimensional species variation onto a few main axes (analogous to principal components analysis), which allowed them to explain much of the variation in crown shape among species using only two free parameters per species. Other researchers [67], [68] have estimated growth and mortality rates for tropical species through Bayesian approaches that structure variation in these demographic rates according to gradients in measured functional traits such as wood density and maximum height. These techniques could be used in conjunction with large inventory datasets to parameterize regional or global versions of our cohort-based canopy model for species-rich forests.

We have demonstrated that the model can accurately predict, and provide a more general understanding of, the growth and yield dynamics of uneven-aged stands. If extended to larger scales, the model could also be used to simulate a broader range of dynamics, such as carbon cycling under different land-use and forest management regimes. Models typically used for this purpose generally do not account for size-related variation in growth and mortality, and therefore may not adequately capture the relationship between stand structure and productivity. However, in scaling from trees to stands our model encompasses only a few of the many levels of biological organization, spanning from physiology to global biogeochemical cycles. Other levels of organization must be incorporated into cohort-based canopy models before they can be used to predict responses to perturbations such as global climate change.

## Supporting Information

Figure S1 **Goodness of fit between predicted and observed tree height for the PB and AR datasets.** The observed and predicted means are plotted for each of seven bins (denoted by the tick marks on the x axis) with the following sample sizes: 64, 714, 1323, 1112, 1105, 985, 99. The line represents a 1∶1 relationship between predicted and observed, and the error bars indicate 1 standard deviation.
(TIFF)

Figure S2 **Goodness of fit between predicted and observed crown depth for the PB and AR datasets.** The observed and predicted means are plotted for each of seven bins (denoted by the tick marks on the x axis) with the following sample sizes: 236, 1039, 901, 771, 760, 816, 865. The line represents a 1∶1 relationship between predicted and observed, and the error bars indicate 1 standard deviation.
(TIFF)

Figure S3 **Goodness of fit between predicted and observed crown radius for the PB and AR datasets.** The observed and predicted means are plotted for each of seven bins (denoted by the tick marks on the x axis) with the following sample sizes: 730, 2785, 1290, 503, 75, 13. The line represents a 1∶1 relationship between predicted and observed, and the error bars indicate 1 standard deviation.
(TIF)

Figure S4 **Goodness of fit between predicted and observed diameter growth.** The observed and predicted means are plotted for each of seven bins (denoted by the tick marks on the x axis) with the following sample sizes: 8959, 5729, 4396, 2151, 667, 156, 46. The line represents a 1∶1 relationship between predicted and observed, and the error bars indicate 1 standard deviation.
(TIFF)

Figure S5 **Goodness of fit between the predicted probability of mortality and observed proportion of dead trees.** The observed and predicted means are plotted for each of 10 bins (denoted by the tick marks on the x axis) with the following sample sizes: 14747, 5342, 1377, 215, 132, 89, 103, 73, 26. The line represents a 1∶1 relationship between predicted and observed.
(TIF)

Figure S6 **Goodness of fit between predicted and observed ingrowth.** The observed and predicted means are plotted for each of five bins (<6, 6–12, 12–18, 18–24, >24) with the following sample sizes: 1906, 198, 77, 30, 29. The line represents a 1∶1 relationship between predicted and observed, and the error bars indicate 1 standard deviation.
(TIFF)

Appendix S1 **Allometric functions.**
(DOCX)

Appendix S2 **Parameter estimation.**
(DOCX)

Appendix S3 **Error distributions.**
(DOCX)

Table S1 **Relative abundance of species by size class, calculated using all three datasets.**
(DOCX)

Table S2 **Maximum likelihood estimates of model parameters (confidence limits in table S3).**
(DOCX)

Table S3 **Confidence intervals of maximum likelihood parameter estimates (see table S2).**
(DOCX)
