# Supplementary figures and images for: How Stand Productivity Results from Size- and Competition-Dependent Growth and Mortality
Source: PLoS One. 2011 Dec 13;6(12):e28660. doi: 10.1371/journal.pone.0028660 (PMC3236764; doi:10.1371/journal.pone.0028660)

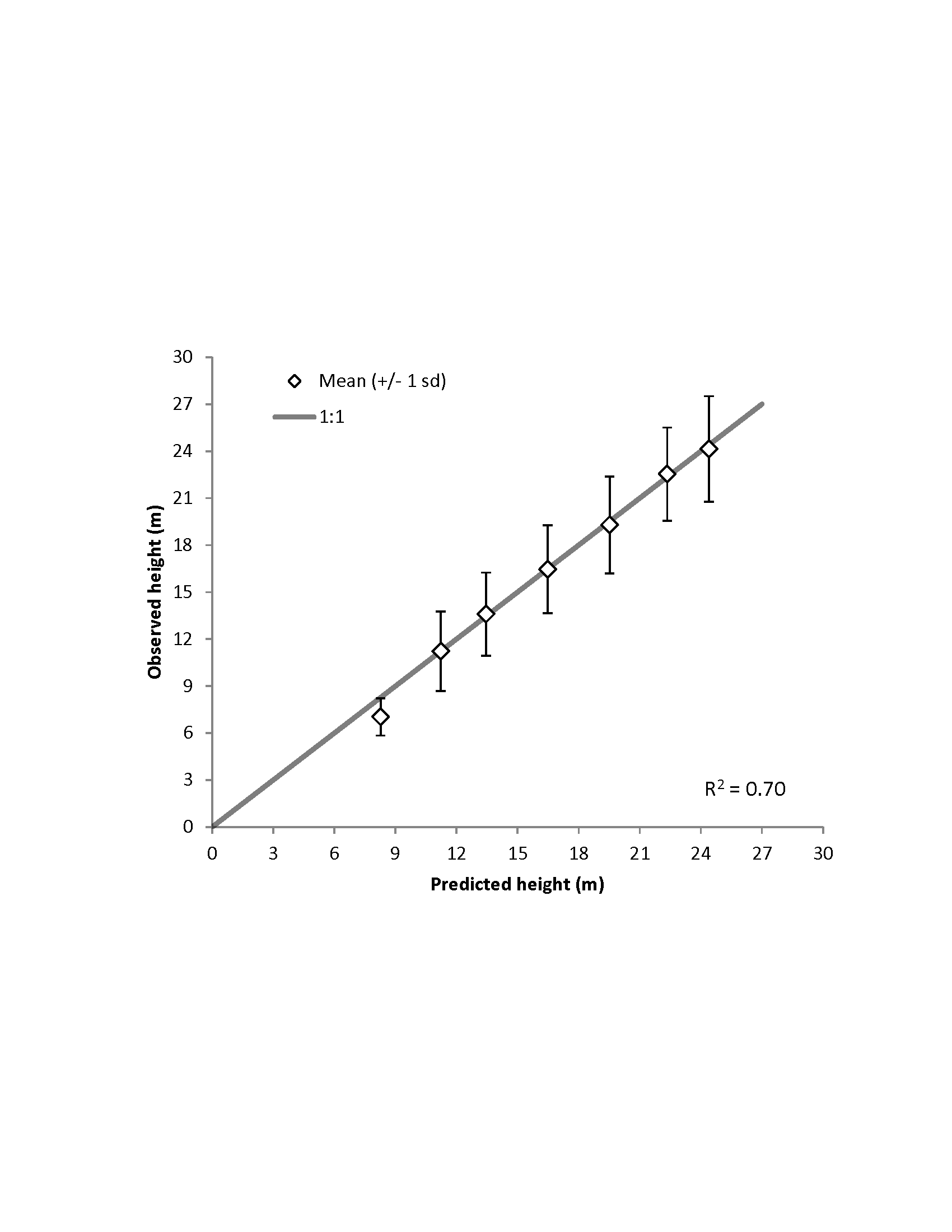

Supplement: Figure S1 — Goodness of fit between predicted and observed tree height for the PB and AR datasets. The observed and predicted means are plotted for each of seven bins (denoted by the tick marks on the x axis) with the following sample sizes: 64, 714, 1323, 1112, 1105, 985, 99. The line represents a 1∶1 relationship between predicted and observed, and the error bars indicate 1 standard deviation. (TIFF) [file pone.0028660.s001.tiff]

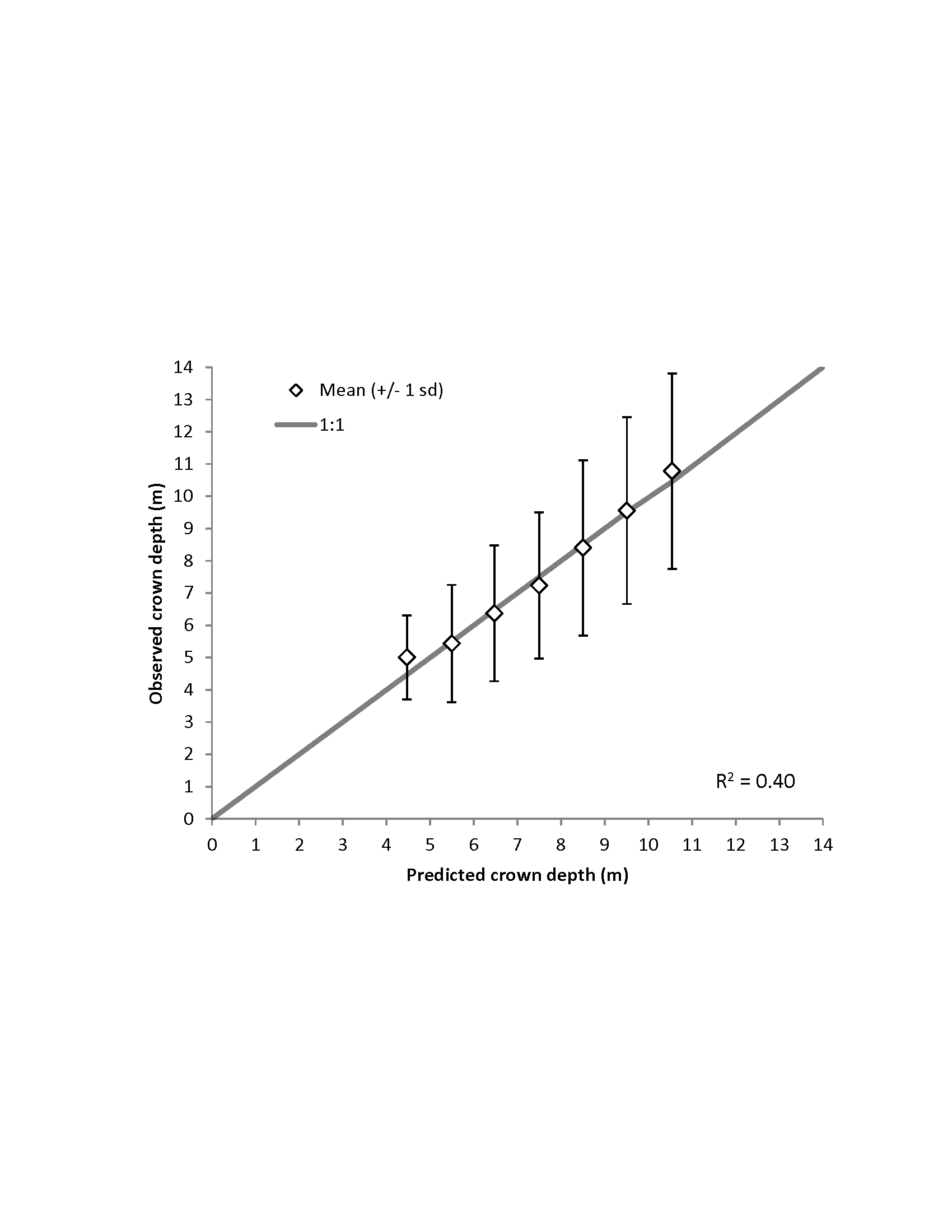

Supplement: Figure S2 — Goodness of fit between predicted and observed crown depth for the PB and AR datasets. The observed and predicted means are plotted for each of seven bins (denoted by the tick marks on the x axis) with the following sample sizes: 236, 1039, 901, 771, 760, 816, 865. The line represents a 1∶1 relationship between predicted and observed, and the error bars indicate 1 standard deviation. (TIFF) [file pone.0028660.s002.tiff]

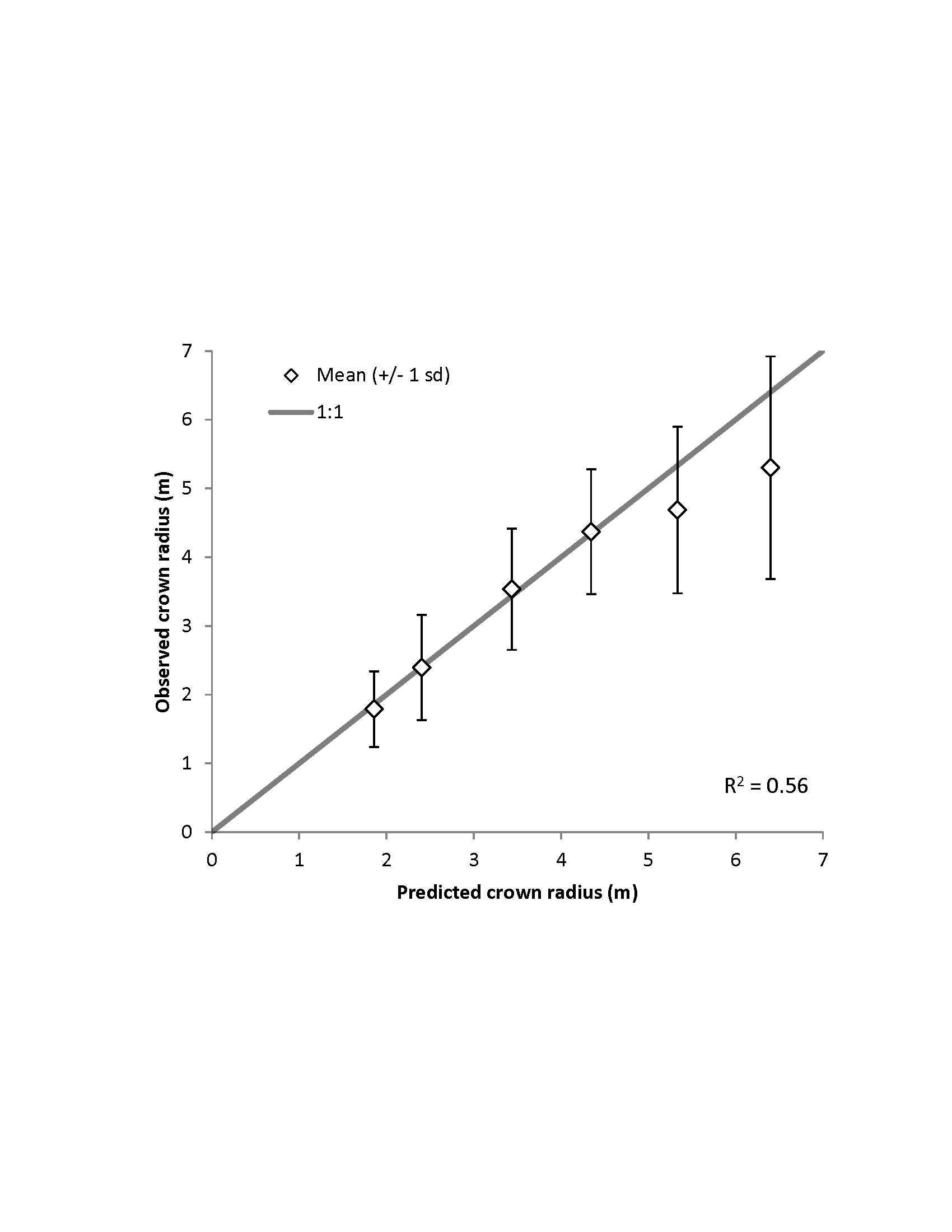

Supplement: Figure S3 — Goodness of fit between predicted and observed crown radius for the PB and AR datasets. The observed and predicted means are plotted for each of seven bins (denoted by the tick marks on the x axis) with the following sample sizes: 730, 2785, 1290, 503, 75, 13. The line represents a 1∶1 relationship between predicted and observed, and the error bars indicate 1 standard deviation. (TIF) [file pone.0028660.s003.tif]

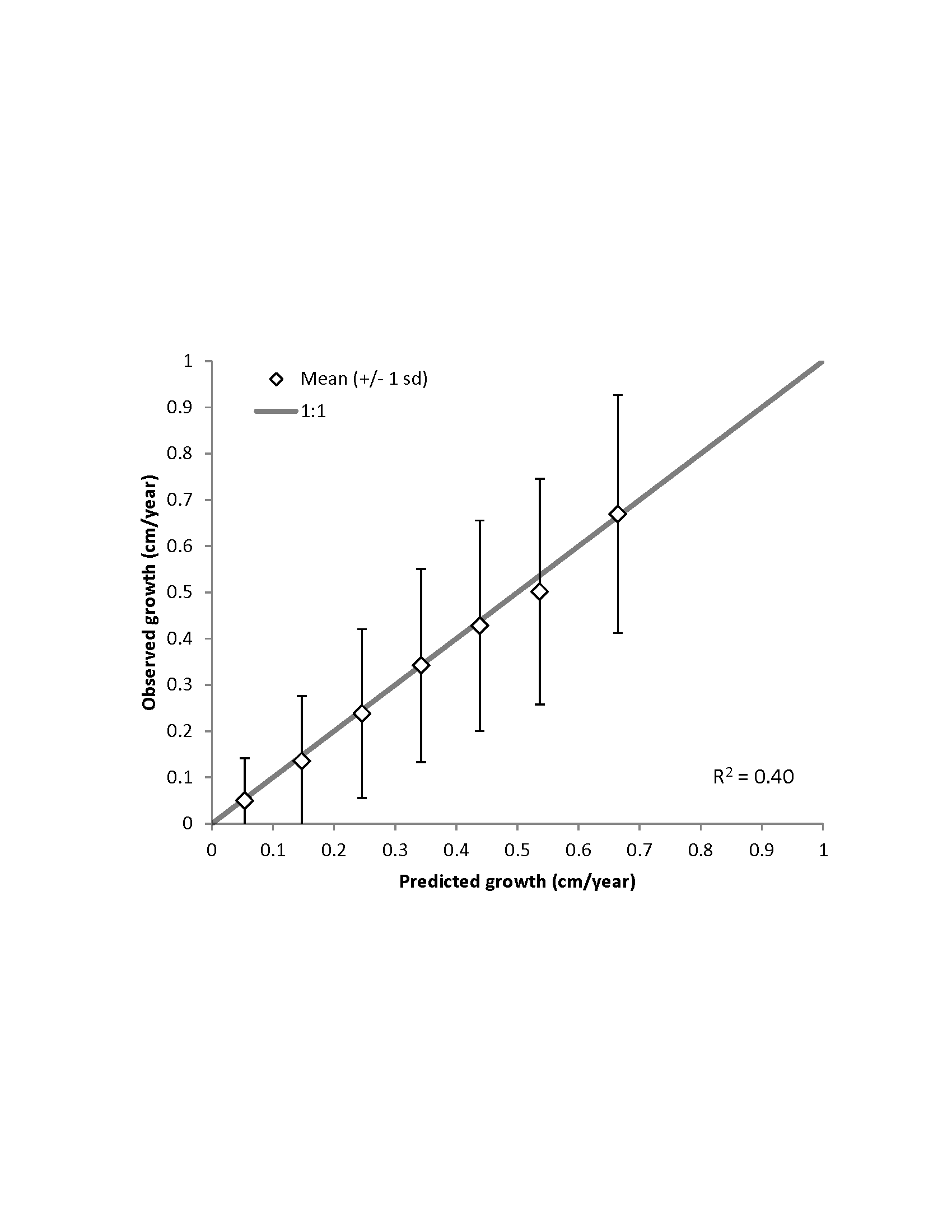

Supplement: Figure S4 — Goodness of fit between predicted and observed diameter growth. The observed and predicted means are plotted for each of seven bins (denoted by the tick marks on the x axis) with the following sample sizes: 8959, 5729, 4396, 2151, 667, 156, 46. The line represents a 1∶1 relationship between predicted and observed, and the error bars indicate 1 standard deviation. (TIFF) [file pone.0028660.s004.tiff]

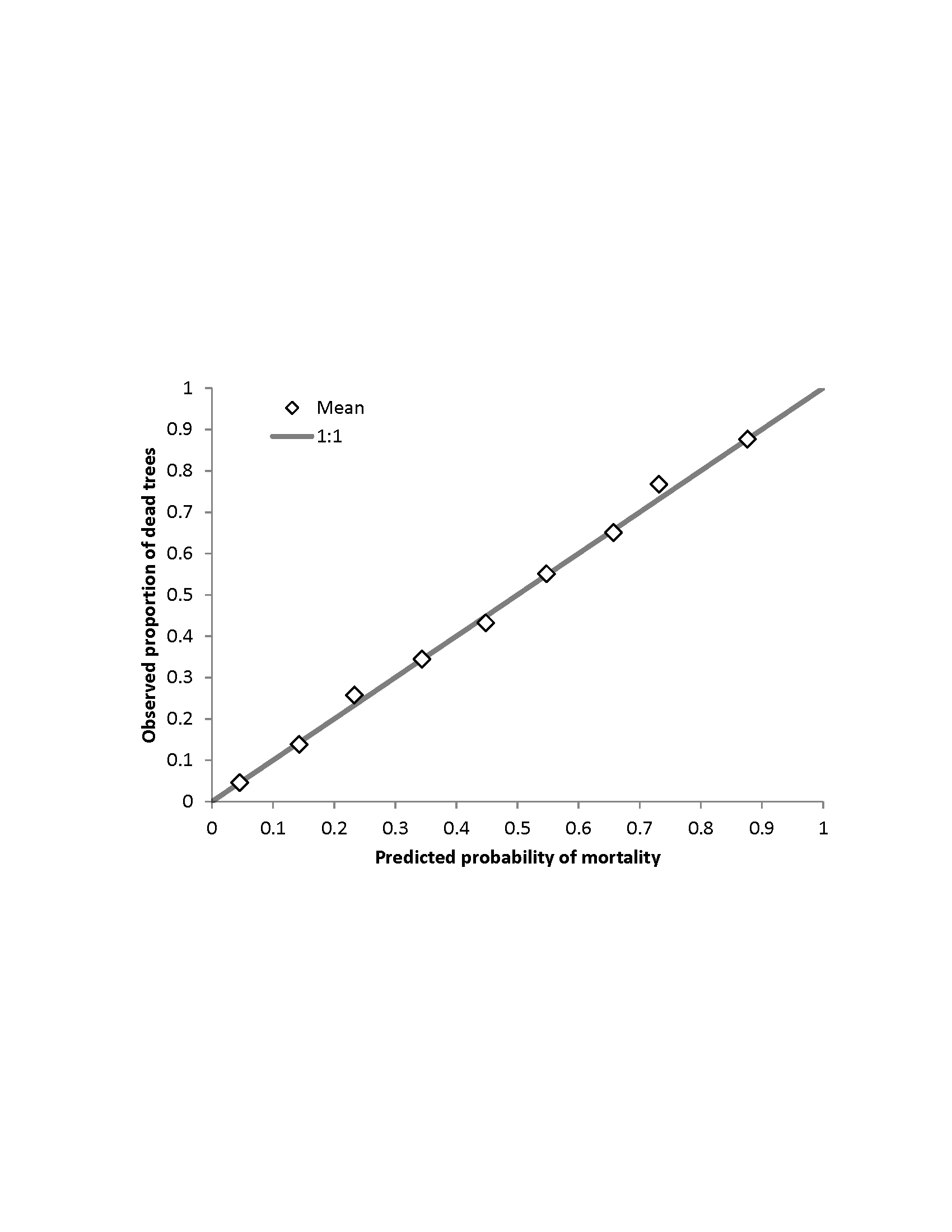

Supplement: Figure S5 — Goodness of fit between the predicted probability of mortality and observed proportion of dead trees. The observed and predicted means are plotted for each of 10 bins (denoted by the tick marks on the x axis) with the following sample sizes: 14747, 5342, 1377, 215, 132, 89, 103, 73, 26. The line represents a 1∶1 relationship between predicted and observed. (TIF) [file pone.0028660.s005.tif]

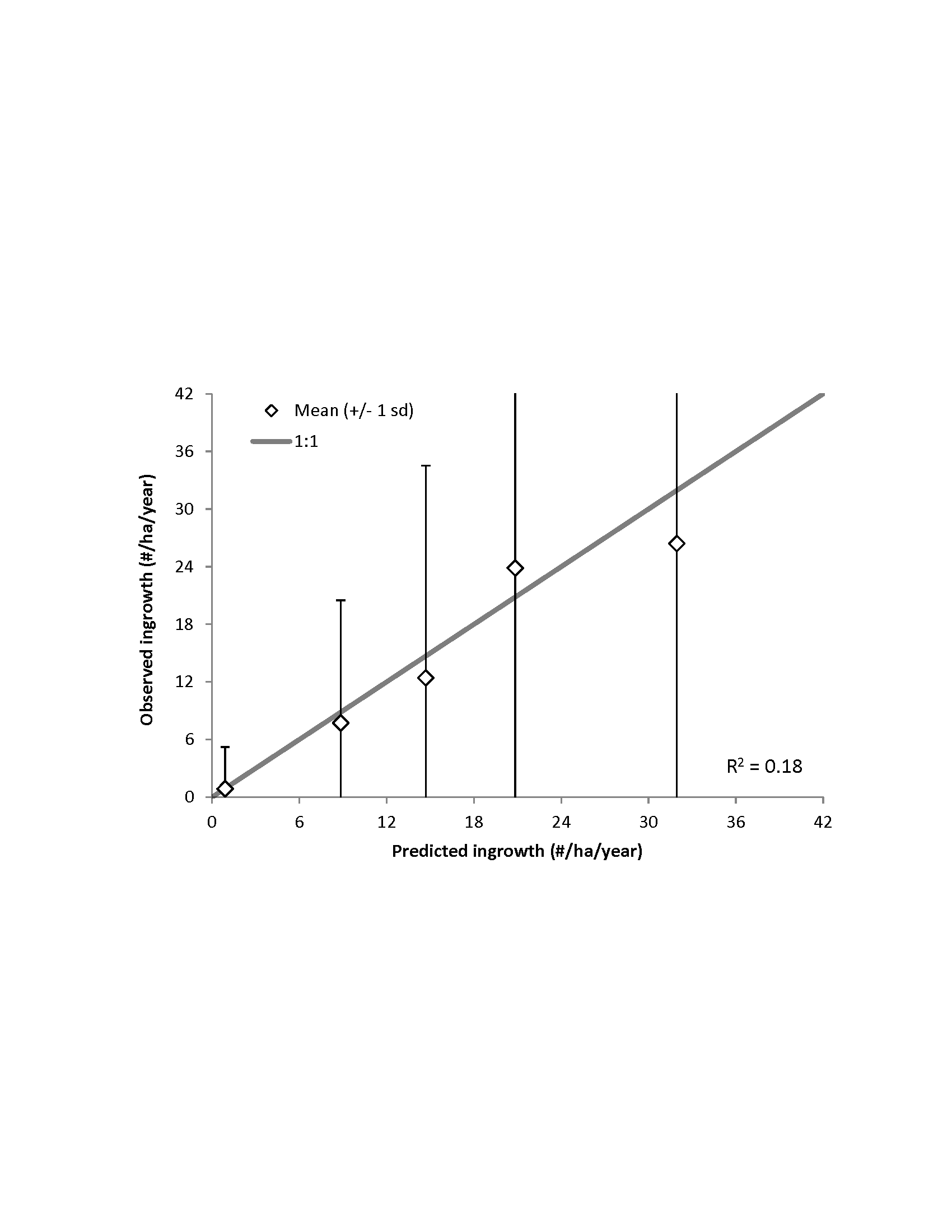

Supplement: Figure S6 — Goodness of fit between predicted and observed ingrowth. The observed and predicted means are plotted for each of five bins (<6, 6–12, 12–18, 18–24, >24) with the following sample sizes: 1906, 198, 77, 30, 29. The line represents a 1∶1 relationship between predicted and observed, and the error bars indicate 1 standard deviation. (TIFF) [file pone.0028660.s006.tiff]
