# Supplementary material for: How Stand Productivity Results from Size- and Competition-Dependent Growth and Mortality
Source: PLoS One. 2011 Dec 13;6(12):e28660. doi: 10.1371/journal.pone.0028660 (PMC3236764; doi:10.1371/journal.pone.0028660)
Supplement: Appendix S1 — Allometric functions. (DOCX) [file pone.0028660.s007.docx]

**Appendix S1: allometric functions**

We modified some of the functional forms in Purves, Lichstein & Pacala (2007) to predict tree height, crown depth, crown radius, and crown area. Here, we describe each of the functional forms and each of the species-specific crown parameters, which are referred to collectively as$\vartheta_{C}$. In addition, we describe the functional form used to predict stem volume.

*Tree height* (*H*) increases asymptotically with diameter:

$H=1.3+(\eta-1.3)\cdot\left(1-e^{\left(\frac{-\phi D}{\eta-1.3}\right)}\right)$ (S1)

where$\phi$ is the initial slope, and $\eta$is the asymptote [A1].

*Crown depth* (*V*) increases linearly with tree height

$V=\varpi H$ (S2)

where $\varpi$ is the ratio of crown depth to tree height (crown ratio), which is assumed to be constant (for reasons discussed further below).

*Mid-crown height* (*h*) is calculated from tree height and crown depth:

$h=H-\left(\frac{V}{2}\right)$ (S3)

*Crown radius* (*R_h_*) at height *h* is:

*R_h_* = $R_{\max}\left(\frac{H-h}{H}\right)^{\beta}$ (S4)

where$\beta$ specifies the shape of the crown (1 = cone, 0 = cylinder) and *R_max_* specifies the maximum crown radius (when $\beta$=0) for a tree of a given diameter:

$R_{\max}=r_{0}+(D/40)\cdot(r_{40}-r_{0})$ (S5)

*Crown area* (*CA*) at the base of the crown is

$CA=\pi R_{H-V}^{^{2}}$ (S6)

and the projected crown area at height *h* from the ground (*CA_h_*) is

$CA_{h}=\pi R_{h}^{2}$ (S7)

Note that the projected area remains constant between the base of the crown and the ground, so *CAI_h_* (eq. 1) is a cumulative metric that increases monotonically from the top to the bottom of the canopy.

All of the functional forms listed above were chosen to provide the best fit to the data (see appendix B). In most cases, the functional forms differ only slightly, if at all, from the original forms in [A2]. However, the original version of the ITD/PPA model had a non-constant crown ratio that could simulate crown lift (a decrease in crown ratio with increasing stand density). We also tried the functional form that was originally used to predict crown ratio, but it did not provide a better fit to the data. This probably reflects that our stands are dominated by relatively shade tolerant species that exhibit limited crown lift.

*Stem volume* (*SV*) is calculated from stem diameter (*D*) and tree height (*H*):

$SV=cD^{2}H$ (S8)

where *c* is a species-specific constant taken from [A3] or derived from [A4]*.* Note that volume increases exponentially with diameter, such that volume growth continues to increase over a range of tree sizes where diameter growth declines (all else being equal).

**References**

S1. Lindner M, Sievanen R, Pretzsch J (1997) Improving the simulation of stand structure in a forest gap model. Forest Ecology and Management 95: 183-195.

S2. Purves DW, Lichstein JW, Pacala SW (2007) Crown plasticity and competition for canopy space: a spatially implicit model parameterized for 250 North American tree species. PLoS ONE 2(9): e870. doi:10.1371/journal.pone.0000870.

S3. Crow TR, Erdmann, GG (1983) Weight and volume equations for the Lake States. Research Paper NC-242, U.S. Dept. of Agriculture, Forest Service, North Central Forest Experiment Station.

S4. Berry AB (1981). Metric form-class volume tables. Information report PI-X-10, Petawawa National Forestry Institute, Canadian Forestry Service, Chalk River, Ontario, Canada.
