# Supplementary material for: How Stand Productivity Results from Size- and Competition-Dependent Growth and Mortality
Source: PLoS One. 2011 Dec 13;6(12):e28660. doi: 10.1371/journal.pone.0028660 (PMC3236764; doi:10.1371/journal.pone.0028660)
Supplement: Appendix S2 — Parameter estimation. (DOCX) [file pone.0028660.s008.docx]

**Appendix S2: parameter estimation**

We used a simulated annealing algorithm to search for the parameter values that maximized the log-likelihood of the data [B1]. The likelihood of the crown data (given the parameters and predictor variables) is:

$L=\prod_{p=1}^{N_{PB,AR}} \prod_{i=1}^{N_{p}} P\left(H_{i},V_{i},W_{i}\left|D_{i},\vartheta_{C},\sigma_{C}\right.\right)$ (S9)

where *p* denotes plots and *i* denotes individual trees, *N_PB,AR_* is the number of plots in the PB and AR datasets, *N_p_* is the number of trees in each plot, *P* denotes probability of the observations for each tree (*H_i_*, *V_i_* and *W_i_*),$\vartheta_{C}$denotes all of the species-specific crown parameters described in appendix A, and $\sigma_{C}$ denotes all of the corresponding species-specific variance parameters described in appendix C.

The likelihood of the demographic data (given the parameters and predictor variables) is:

$$L=$$

$$\prod_{p=1}^{N_{PB,AR,PSP}} \prod_{i=1}^{N_{p}} P\left(\Delta D_{i}\left|D_{i},\overline{H}_{i},\overline{CA}_{i},\overline{CAI}_{h,i},\vartheta_{\Delta D},\sigma_{\Delta D}\right.\right)\bullet$$

$$\prod_{p=1}^{N_{PB,AR,PSP}} \prod_{i=1}^{N_{p}} P\left(M_{i}\left|D_{i},\overline{H}_{i},\overline{CA}_{i},\overline{CAI}_{h,i},\vartheta_{M}\right.\right)\bullet$$

$$\prod_{p=1}^{N_{PSP}} \prod_{s=1}^{8} P\left(I_{p}\left|S,\overline{CAI_{p}},\vartheta_{I},\sigma_{I}\right.\right)\bullet$$

$$\prod_{e=1}^{N_{SE}} P\left(E_{e}\left|\sigma_{E}\right.\right)$$

(S10)

where $\Delta D_{i}$is the observed diameter growth rate of tree *i*, *M_i_* is the observed status (live or dead) upon remeasurement, *I* is the ingrowth for species *s* (whose presence is indicated by S), E_e_ are the stand-effect multipliers for the N_SE_ stands in the datasets, $\vartheta_{\Delta D,M,I}$ are the corresponding species-specific demographic parameters in equations 1-9, and$\sigma_{\Delta D,I,E}$ are the corresponding variance parameters of the error distributions described in appendix C. The recruitment model was only fit to the PSP dataset because it had the lowest threshold for inventorying trees (2.5 cm in diameter).

Confidence limits were computed by repeatedly sampling random parameter values to obtain 100,000 sets of parameters. We then calculated the log-likelihood of each parameter set and its deviance from the maximum log-likelihood (D = 2(*L - L_max_*)). Sets for which the deviance exceeded the critical value of the $\chi^{2}$distribution ($\alpha$= 0.05, df = 1) were excluded. From the remaining sets, we selected the minimum and maximum parameter values as the 95% confidence limits.

**References**

S5. Kirkpatrick S, Gelatt CD Jr, Vecchi MP (1983) Optimization by simulated annealing. Science 220: 671-680.
