# Supplementary material for: How Stand Productivity Results from Size- and Competition-Dependent Growth and Mortality
Source: PLoS One. 2011 Dec 13;6(12):e28660. doi: 10.1371/journal.pone.0028660 (PMC3236764; doi:10.1371/journal.pone.0028660)
Supplement: Appendix S3 — Error distributions. (DOCX) [file pone.0028660.s009.docx]

**Appendix S3: error distributions**

Here, we describe the error distribution of each of the observed variables (denoted here with the subscript *o*), including the species-specific variance parameters, referred to collectively as$\sigma_{C}$ (crown parameters), $\sigma_{\Delta D}$ (growth parameters), and $\sigma_{I}$ (ingrowth parameters). We also describe the distribution we used to estimate random stand effects for growth, mortality, and ingrowth.

*Tree height, crown depth and width* (*H*, *V* and *W*). We assumed that *H_o_*, *V_o_* and *W_o_* follow a multivariate normal distribution:

$P(H_{o},V_{o},W_{o}/2)=N(\mu,\Sigma)$ (S11)

where

$\mu=\left\{H,V,R_{H-V}\right\}$ (S12)

$\Sigma=\left[\begin{matrix} \sigma_{H}^{2} & \rho_{HV}\sigma_{H}\sigma_{V} & \rho_{HW}\sigma_{H}\sigma_{W} \\ \rho_{HV}\sigma_{H}\sigma_{V} & \sigma_{V}^{2} & \rho_{VW}\sigma_{V}\sigma_{W} \\ \rho_{HW}\sigma_{H}\sigma_{W} & \rho_{VW}\sigma_{V}\sigma_{W} & \sigma_{W}^{2} \end{matrix}\right]$ (S13)

*Diameter growth* ($G$). We assumed that $G_{o}$/ *y* follows a normal distribution:

$P(G_{0}/y)=N(G,\sigma_{G})$ (S14)

where $G_{o}$is the difference in tree diameter between measurements (or 0, if the second diameter measurement was smaller than the first), and *y* is the interval between measurements, in years.

*Mortality* (*M*). We assumed that *M_o_* follows a Bernouilli distribution:

$1-(1-M)^{y}$, if *M_o_* = 0

$P(M_{o})=$ (S15)

$(1-M)^{y}$, if *M_o_* = 1

*Ingrowth* (*I*). We assumed that *I_o_* follows a negative binomial distribution (parameterized by its expected value and an overdispersion parameter Ω):

$NB(I\cdot y\cdot0.04,\Omega_{P})$, if the species is present

$P(I_{0})=$ (S16)

$NB(I\cdot y\cdot0.04,\Omega_{A})$, if the species is absent

where *I* is the expected rate of ingrowth (eq. 7), *I_o_* is the observed rate of ingrowth, and $\Omega_{P}$is the overdispersion parameter for stands in which the species is already present, and $\Omega_{A}$for stands in which it is not.

*Stand effects (E).* We assumed that the logarithms of stand effects followed a normal distribution with a mean of 0:

$P(\ln(E))=N(0,\sigma_{E})$ (S17)
