# Supplementary material for: How Stand Productivity Results from Size- and Competition-Dependent Growth and Mortality
Source: PLoS One. 2011 Dec 13;6(12):e28660. doi: 10.1371/journal.pone.0028660 (PMC3236764; doi:10.1371/journal.pone.0028660)
Supplement: Table S1 — Relative abundance of species by size class, calculated using all three datasets. (DOCX) [file pone.0028660.s010.docx]

**Table S1.** Relative abundance of species by size class, calculated using all three datasets.

| **Diameter** | **Sugar** | **Beech** | **Yellow** | **Ironwood** | **Eastern** | **White** | **Red** | **Basswood** |
| --- | --- | --- | --- | --- | --- | --- | --- | --- |
| **class** | **maple** |  | **birch** |  | **hemlock** | **ash** | **maple** |  |
| **5-10** | 0.63 | 0.011 | 0.086 | 0.162 | 0.01 | 0.049 | 0.014 | 0.039 |
| **10-15** | 0.681 | 0.033 | 0.067 | 0.142 | 0.007 | 0.027 | 0.012 | 0.03 |
| **15-20** | 0.763 | 0.052 | 0.032 | 0.067 | 0.009 | 0.039 | 0.011 | 0.027 |
| **20-25** | 0.803 | 0.061 | 0.018 | 0.024 | 0.011 | 0.036 | 0.016 | 0.031 |
| **25-30** | 0.815 | 0.067 | 0.023 | 0.008 | 0.02 | 0.019 | 0.021 | 0.027 |
| **30-35** | 0.756 | 0.098 | 0.036 | 0.007 | 0.036 | 0.019 | 0.018 | 0.031 |
| **35-40** | 0.76 | 0.088 | 0.037 | 0.004 | 0.048 | 0.012 | 0.013 | 0.036 |
| **40-45** | 0.747 | 0.093 | 0.047 | 0.004 | 0.058 | 0.006 | 0.01 | 0.035 |
| **45-50** | 0.793 | 0.07 | 0.047 | 0 | 0.031 | 0.008 | 0.02 | 0.031 |
| **50-55** | 0.72 | 0.113 | 0.1 | 0 | 0.047 | 0 | 0.007 | 0.013 |
| **>55** | 0.805 | 0.049 | 0.085 | 0 | 0.024 | 0.012 | 0 | 0.024 |
