# Supplementary material for: How Stand Productivity Results from Size- and Competition-Dependent Growth and Mortality
Source: PLoS One. 2011 Dec 13;6(12):e28660. doi: 10.1371/journal.pone.0028660 (PMC3236764; doi:10.1371/journal.pone.0028660)
Supplement: Table S2 — Maximum likelihood estimates of model parameters (confidence limits in table S3). (DOCX) [file pone.0028660.s011.docx]

**Table S2.** Maximum likelihood estimates of model parameters (confidence limits in table S3)

| **Model** | **Parameter** | **Sugar** | **Beech** | **Yellow** | **Ironwood** | **Eastern** | **White** | **Red** | **Basswood** | **All** |
| --- | --- | --- | --- | --- | --- | --- | --- | --- | --- | --- |
|  |  | **maple** |  | **birch** |  | **hemlock** | **ash** | **maple** |  | **species** |
| **Height allometry (H)** | η | 25.3588 | 23.8095 | 24.7702 | 19.0506 | 26.9651 | 25.0638 | 20.9017 | 30.4785 |  |
|  | ϕ | 1.35017 | 1.29675 | 1.19439 | 1.5723 | 0.72939 | 1.44406 | 1.65906 | 1.20491 |  |
| **Crown depth (V)** | ϖ | 0.46099 | 0.47493 | 0.45541 | 0.43857 | 0.52407 | 0.36298 | 0.40956 | 0.40127 |  |
| **Crown radius (R_h_)** | β | 0.22977 | 1.04203 | 1.03778 | 0.3837 | 1.33787 | 0.0001 | 0.0001 | 0.75358 |  |
|  | r_0_ | 1.52529 | 2.41848 | 4.1811 | 1.44703 | 1.55475 | 1.19352 | 1.04395 | 1.04292 |  |
|  | r_40_ | 4.94851 | 9.0831 | 8.09932 | 5.48292 | 8.24872 | 3.47522 | 4.03818 | 8.15148 |  |
| **Growth (G)** | δ | 0.61109 | 0.52551 | 1.30557 | 2.18834 | 0.44978 | 0.77378 | 0.56784 | 0.41304 |  |
|  | γ | 16.3733 | 15.5299 | 5.33962 | 0.34532 | 0.00351 | 4.6144 | 27.0681 | 96.2306 |  |
|  | ν | 1.33027 | 1.49276 | 1.41861 | 1.89508 | 17.1927 | 2.09187 | 1.95485 | 4.48802 |  |
|  | ζ | 0.04334 | 0.13417 | 0.001 | 0.01229 | 0.23636 | 0.001 | 0.001 | 0.001 |  |
|  | κ | 0.94115 | 1.20622 | 1.19537 | 1.20353 | 0.87344 | 1.0326 | 0.854 | 0.79877 |  |
| **Mortality (M)** | ψ | 124.65 | 43.6635 | 1000 | 61.3378 | 1000 | 13.5761 | 831.227 | 1000 |  |
|  | Φ | 0.52949 | 0.88689 | 0.03669 | 0.92197 | 0.90426 | 1.3064 | 0.57402 | 0.28568 |  |
|  | θ | 0.43516 | 0.25 | 0.48381 | 0.25 | 0.25 | 0.25 | 0.75 | 0.68633 |  |
|  | D_0.01_ | 85.173 | 64.9 | 83.3 | 38.6 | 55.4 | 55.9 | 51.4 | 60.4 |  |
|  | ω | 0.10131 | 0.001 | 0.00316 | 0.001 | 0.08834 | 0.001 | 0.00113 | 0.00904 |  |
|  | ο | 1.6382 | 0.12226 | 1.85522 | 0.38459 | 4.29049 | 0.47653 | 2.90426 | 3.65333 |  |
| **Ingrowth (I)** | τ | 26.5196 | 5.91306 | 14.6688 | 23.1297 | 6.87007 | 8.1245 | 11.1492 | 7.8122 |  |
|  | χ | 0.20392 | 0.48317 | 0.0313 | 0.0001 | 0.0001 | 0.0001 | 0.03931 | 0.0001 |  |
|  | υ | 1.6863 | 20 | 2.07292 | 0.92588 | 0.86997 | 1.15942 | 6.73676 | 1.11814 |  |
|  | CAI_0.05_ | 1.28185 | 1.4866 | 1.3976 | 1.4674 | 1.4599 | 1.4713 | 1.4978 | 1.4712 |  |
|  | ξ | n/a | 0.29779 | 0.45099 | 1.63507 | 0.14247 | 0.5562 | 0.09784 | 0.0521 |  |
| **Error distributions** | σ_H_ | 2.73997 | 2.70717 | 2.95072 | 2.071 | 2.63663 | 1.42716 | 3.33172 | 3.23495 |  |
|  | σ_V_ | 2.4497 | 2.49678 | 2.47505 | 1.70195 | 2.89641 | 0.46474 | 2.08304 | 2.55426 |  |
|  | σ_W_ | 0.75117 | 0.84294 | 0.83178 | 0.60013 | 0.64463 | 0.38651 | 0.69272 | 0.69631 |  |
|  | ρ_HV_ | 0.4692 | 0.56109 | 0.62716 | 0.19236 | 0.5438 | 0.35538 | 0.47311 | 0.4695 |  |
|  | ρ_HW_ | 0.03269 | 0.02845 | 0.23835 | -0.04367 | 0.01439 | -0.1252 | 0.00369 | 0.02874 |  |
|  | ρ_VW_ | 0.06443 | -4.4E-05 | 0.47298 | 0.23291 | 0.1278 | -0.2222 | 0.04838 | 0.07946 |  |
|  | σ_G_ | 0.13409 | 0.15876 | 0.14173 | 0.07338 | 0.14643 | 0.10545 | 0.14884 | 0.12923 |  |
|  | Ω_P_ | 0.49215 | 0.29137 | 0.14836 | 0.12083 | 0.45172 | 0.47713 | 0.4315 | 0.38847 |  |
|  | Ω_A_ | n/a | 0.00301 | 0.03329 | 0.04277 | 0.00915 | 0.01848 | 0.02631 | 5.65749 |  |
| **Stand effects (E)** | α |  |  |  |  |  |  |  |  | 0.13502 |
|  | π |  |  |  |  |  |  |  |  | 1.71789 |
| **Stand error structure** | σ_E_ |  |  |  |  |  |  |  |  | 0.25993 |
