# Supplementary material for: How Stand Productivity Results from Size- and Competition-Dependent Growth and Mortality
Source: PLoS One. 2011 Dec 13;6(12):e28660. doi: 10.1371/journal.pone.0028660 (PMC3236764; doi:10.1371/journal.pone.0028660)
Supplement: Table S3 — Confidence intervals of maximum likelihood parameter estimates (see table S2). (DOCX) [file pone.0028660.s012.docx]

Table S3. Confidence intervals of maximum likelihood parameter estimates (see table S2)

| **Model** | **Parameter** | **Sugar maple** | | **Beech** | | **Yellow birch** | | **Ironwood** | |
| --- | --- | --- | --- | --- | --- | --- | --- | --- | --- |
|  |  | Lower | Upper | Lower | Upper | Lower | Upper | Lower | Upper |
| **Height allometry (H)** | η | 25.2651 | 25.4421 | 23.6162 | 24.013 | 24.3362 | 25.3302 | 18.6261 | 19.4916 |
|  | ϕ | 1.34467 | 1.35479 | 1.28351 | 1.31437 | 1.14311 | 1.23393 | 1.53716 | 1.60207 |
| **Crown depth (V)** | ϖ | 0.45969 | 0.4619 | 0.47211 | 0.47726 | 0.45016 | 0.46208 | 0.43351 | 0.44383 |
| **Crown radius (R_h_)** | β | 0.22255 | 0.23762 | 1.01849 | 1.06318 | 0.98285 | 1.07811 | 0.36194 | 0.41123 |
|  | r_0_ | 1.50483 | 1.55158 | 2.31153 | 2.54481 | 3.92218 | 4.41847 | 1.36581 | 1.53057 |
|  | r_40_ | 4.91789 | 4.97733 | 8.87821 | 9.18623 | 7.79143 | 8.33067 | 5.30517 | 5.67222 |
| **Growth (G)** | δ | 0.61107 | 0.61109 | 0.50952 | 0.53677 | 1.27836 | 1.30993 | 2.16833 | 2.21201 |
|  | γ | 16.3724 | 16.3741 | 14.3889 | 15.9977 | 5.22189 | 5.40945 | 0.33992 | 0.34925 |
|  | ν | 1.33022 | 1.33031 | 1.46112 | 1.60438 | 1.40513 | 1.43906 | 1.88966 | 1.89907 |
|  | ζ | 0.04332 | 0.04335 | 0.12549 | 0.14782 | 0.001 | 0.00281 | 0.01106 | 0.01581 |
|  | κ | 0.94115 | 0.94112 | 1.22939 | 1.16054 | 1.20359 | 1.17886 | 1.21357 | 1.19439 |
| **Mortality (M)** | ψ | 118.904 | 129.463 | 35.1084 | 46.3478 | 950.107 | 1000 | 54.051 | 66.2877 |
|  | Φ | 0.52474 | 0.55466 | 0.85168 | 0.94589 | 0.01299 | 0.07545 | 0.87103 | 0.96629 |
|  | θ | 0.40649 | 0.45262 | 0.25 | 0.27927 | 0.40058 | 0.54785 | 0.25 | 0.25188 |
|  | D_0.01_ | 82.7279 | 87.0063 | n/a | n/a | n/a | n/a | n/a | n/a |
|  | ω | 0.0977 | 0.10579 | 0.001 | 0.78638 | 0.00285 | 0.00342 | 0.001 | 0.0423 |
|  | ο | 1.69777 | 1.6045 | 0.29153 | 0.01603 | 1.88105 | 1.81797 | 0.42354 | 0.34749 |
| **Recruitment (I_S_)** | τ | 24.5969 | 28.4407 | 4.39103 | 7.30032 | 11.9697 | 18.4093 | 18.0928 | 27.1131 |
|  | χ | 0.17619 | 0.24607 | 0.38333 | 0.6145 | 0.01476 | 0.05293 | 0.0001 | 0.01214 |
|  | υ | 1.51155 | 1.94 | 1.92039 | 20 | 1.74169 | 2.38586 | 0.77613 | 1.0595 |
|  | CAI_0.05_ | n/a | n/a | n/a | n/a | n/a | n/a | n/a | n/a |
|  | ξ | n/a | n/a | 0.13824 | 0.65359 | 0.33525 | 0.57643 | 1.13803 | 2.29116 |
| **Error distributions** | σ_H_ | 2.71836 | 2.76016 | 2.6671 | 2.74992 | 2.823 | 3.07661 | 2.01808 | 2.13252 |
|  | σ_V_ | 2.43253 | 2.46618 | 2.4582 | 2.5416 | 2.36336 | 2.59539 | 1.6576 | 1.73671 |
|  | σ_W_ | 0.74543 | 0.75609 | 0.82946 | 0.85926 | 0.80273 | 0.86779 | 0.58732 | 0.6173 |
|  | ρ_HV_ | 0.46122 | 0.4773 | 0.54369 | 0.57709 | 0.59931 | 0.66507 | 0.15988 | 0.22165 |
|  | ρ_HW_ | 0.02583 | 0.03933 | 0.00885 | 0.04713 | 0.19649 | 0.2834 | -0.07784 | -0.01898 |
|  | ρ_VW_ | 0.0567 | 0.07492 | -0.02849 | 0.0258 | 0.41467 | 0.52449 | 0.20154 | 0.25925 |
|  | σ_G_ | 0.13409 | 0.1341 | 0.1577 | 0.16083 | 0.14004 | 0.14328 | 0.07305 | 0.07401 |
|  | Ω_P_ | 0.45588 | 0.52193 | 0.21504 | 0.40016 | 0.12291 | 0.18076 | 0.10722 | 0.1349 |
|  | Ω_A_ | n/a | n/a | 0.00203 | 0.00442 | 0.02558 | 0.04614 | 0.03468 | 0.05409 |

Table S3, continued. Confidence intervals of maximum likelihood parameter estimates (see table S2)

| **Model** | **Parameter** | **Hemlock** | | **White ash** | | **Red maple** | | **Basswood** | | **All species** | |
| --- | --- | --- | --- | --- | --- | --- | --- | --- | --- | --- | --- |
|  |  | Lower | Upper | Lower | Upper | Lower | Upper | Lower | Upper | Lower | Upper |
| **Height allometry (H)** | η | 25.8911 | 27.8908 | 24.2954 | 25.8651 | 20.2315 | 21.4944 | 29.1773 | 31.7178 |  |  |
|  | ϕ | 0.71046 | 0.75505 | 1.38177 | 1.49283 | 1.56883 | 1.77272 | 1.16382 | 1.24488 |  |  |
| **Crown depth (V)** | ϖ | 0.51861 | 0.53189 | 0.35727 | 0.36884 | 0.40339 | 0.41678 | 0.39412 | 0.41268 |  |  |
| **Crown radius (R_h_)** | β | 1.28702 | 1.37687 | 0.0001 | 0.0001 | 0.0001 | 0.0001 | 0.72285 | 0.78968 |  |  |
|  | r_0_ | 1.3256 | 1.8256 | 1.0502 | 1.33461 | 0.88105 | 1.15198 | 0.82524 | 1.34775 |  |  |
|  | r_40_ | 8.01792 | 8.49993 | 3.3323 | 3.61755 | 3.92248 | 4.15271 | 7.83374 | 8.48127 |  |  |
| **Growth (G)** | δ | 0.42351 | 0.4683 | 0.76068 | 0.79223 | 0.52938 | 0.58267 | 0.4033 | 0.41868 |  |  |
|  | γ | 0.00096 | 0.00511 | 4.22954 | 4.92237 | 21.415 | 29.492 | 86.1984 | 100 |  |  |
|  | ν | 14.3529 | 19.5839 | 2.01374 | 2.15292 | 1.6144 | 2.01474 | 4.31567 | 5.3922 |  |  |
|  | ζ | 0.19136 | 0.25877 | 0.001 | 0.00645 | 0.001 | 0.03478 | 0.001 | 0.01545 |  |  |
|  | κ | 0.88675 | 0.75467 | 1.04789 | 1.0226 | 0.87784 | 0.79123 | 0.84195 | 0.78569 |  |  |
| **Mortality (M)** | ψ | 747.24 | 1000 | 12.7623 | 14.8095 | 685.173 | 964.372 | 849.343 | 1000 |  |  |
|  | Φ | 0.82817 | 1.01928 | 1.26332 | 1.34083 | 0.53144 | 0.67091 | 0.24604 | 0.34853 |  |  |
|  | θ | 0.25 | 0.25943 | 0.25 | 0.27235 | 0.68439 | 0.75 | 0.60823 | 0.75 |  |  |
|  | D_0.01_ | n/a | n/a | n/a | n/a | n/a | n/a | n/a | n/a |  |  |
|  | ω | 0.06731 | 0.13587 | 0.001 | 0.0193 | 0.001 | 0.00142 | 0.00853 | 0.01042 |  |  |
|  | ο | 5.72478 | 3.30323 | 0.51797 | 0.44625 | 2.98824 | 2.7636 | 3.87591 | 3.43392 |  |  |
| **Recruitment (I)** | τ | 5.07538 | 9.46829 | 6.7381 | 10.4569 | 8.09905 | 14.8658 | 6.82197 | 9.29288 |  |  |
|  | χ | 0.0001 | 0.11085 | 0.0001 | 0.03067 | 0.02204 | 0.06283 | 0.0001 | 0.01888 |  |  |
|  | υ | 0.54027 | 1.15121 | 0.90085 | 1.37756 | 5.23617 | 8.74924 | 0.95478 | 1.31711 |  |  |
|  | CAI_0.05_ | n/a | n/a | n/a | n/a | n/a | n/a | n/a | n/a |  |  |
|  | ξ | 0.09363 | 0.20849 | 0.38567 | 0.79254 | 0.06322 | 0.13654 | 0.03512 | 0.08182 |  |  |
| **Error distributions** | σ_H_ | 2.51915 | 2.73282 | 1.2863 | 1.63345 | 3.144 | 3.50996 | 3.07545 | 3.40659 |  |  |
|  | σ_V_ | 2.80616 | 2.99949 | 0.41143 | 0.54339 | 1.97695 | 2.17463 | 2.41043 | 2.68621 |  |  |
|  | σ_W_ | 0.62065 | 0.67306 | 0.34957 | 0.44642 | 0.66591 | 0.72472 | 0.66322 | 0.73022 |  |  |
|  | ρ_HV_ | 0.51028 | 0.57746 | 0.14942 | 0.48591 | 0.40393 | 0.52865 | 0.40104 | 0.52688 |  |  |
|  | ρ_HW_ | -0.03188 | 0.07091 | -0.23278 | 0.03579 | -0.05756 | 0.05013 | -0.02551 | 0.08147 |  |  |
|  | ρ_VW_ | 0.07346 | 0.20046 | -0.38258 | -0.06145 | -0.01937 | 0.11905 | 0.0135 | 0.14147 |  |  |
|  | σ_G_ | 0.14335 | 0.15024 | 0.10416 | 0.10683 | 0.14551 | 0.15238 | 0.12673 | 0.1306 |  |  |
|  | Ω_P_ | 0.28154 | 0.65714 | 0.34405 | 0.62061 | 0.27601 | 0.74571 | 0.31028 | 0.50334 |  |  |
|  | Ω_A_ | 0.006 | 0.01541 | 0.01405 | 0.02464 | 0.01421 | 0.05092 | 0.11871 | 10 |  |  |
| **Stand effects (E)** | α |  |  |  |  |  |  |  |  | 0.086 | 0.165 |
|  | π |  |  |  |  |  |  |  |  | 1.575 | 1.872 |
| **Stand error structure** | σ_E_ |  |  |  |  |  |  |  |  | 0.255 | 0.263 |
